# A Single Nucleotide Substitution of *GSAM* Gene Causes Massive Accumulation of Glutamate 1-Semialdehyde and Yellow Leaf Phenotype in Rice

**DOI:** 10.1186/s12284-021-00492-x

**Published:** 2021-06-05

**Authors:** Qian Wang, Baiyang Zhu, Congping Chen, Zhaodi Yuan, Jia Guo, Xiaorong Yang, San Wang, Yan Lv, Qingsong Liu, Bin Yang, Changhui Sun, Pingrong Wang, Xiaojian Deng

**Affiliations:** 1 State Key Laboratory of Crop Gene Exploration and Utilization in Southwest China, Sichuan Agricultural University, Chengdu, 611130 China; 2 Rice Research Institute, Sichuan Agricultural University, Chengdu, 611130 China

**Keywords:** Rice, Tetrapyrrol biosynthesis, *GSAM* gene, Protein interaction, Chloroplast development, Yellow leaf mutant, Gene cloning

## Abstract

**Background:**

Tetrapyrroles play indispensable roles in various biological processes. In higher plants, glutamate 1-semialdehyde 2,1-aminomutase (GSAM) converts glutamate 1-semialdehyde (GSA) to 5-aminolevulinic acid (ALA), which is the rate-limiting step of tetrapyrrole biosynthesis. Up to now, *GSAM* genes have been successively identified from many species. Besides, it was found that GSAM could form a dimeric protein with itself by x-ray crystallography. However, no mutant of *GSAM* has been identified in monocotyledonous plants, and no experiment on interaction of GSAM protein with itself has been reported so far.

**Result:**

We isolated a yellow leaf mutant, *ys53*, in rice (*Oryza sativa*). The mutant showed decreased photosynthetic pigment contents, suppressed chloroplast development, and reduced photosynthetic capacity. In consequence, its major agronomic traits were significantly affected. Map-based cloning revealed that the candidate gene was *LOC_Os08g41990* encoding GSAM protein. In *ys53* mutant, a single nucleotide substitution in this gene caused an amino acid change in the encoded protein, so its ALA-synthesis ability was significantly reduced and GSA was massively accumulated. Complementation assays suggested the mutant phenotype of *ys53* could be rescued by introducing wild-type Os*GSAM* gene, confirming that the point mutation in *OsGSAM* is the cause of the mutant phenotype. *OsGSAM* is mainly expressed in green tissues, and its encoded protein is localized to chloroplast. qRT-PCR analysis indicated that the mutation of *OsGSAM* not only affected the expressions of tetrapyrrole biosynthetic genes, but also influenced those of photosynthetic genes in rice. In addition, the yeast two-hybrid experiment showed that OsGSAM protein could interact with itself, which could largely depend on the two specific regions containing the 81th–160th and the 321th–400th amino acid residues at its N- and C-terminals, respectively.

**Conclusions:**

We successfully characterized rice *GSAM* gene by a yellow leaf mutant and map-based cloning approach. Meanwhile, we verified that OsGSAM protein could interact with itself mainly by means of the two specific regions of amino acid residues at its N- and C-terminals, respectively.

**Supplementary Information:**

The online version contains supplementary material available at 10.1186/s12284-021-00492-x.

## Background

Tetrapyrroles play vital roles in various key biological processes, including photosynthesis and respiration (Ilag et al. 1994; Mochizuki et al. 2010). There are four classes of tetrapyrroles in higher plants, namely, chlorophyll (Chl), heme, siroheme, and phytochromobilin (Tanaka et al. 2011). All of these tetrapyrroles are derived from a common biosynthetic pathway that resides in the plastid, in which eight molecules of 5-aminolevulinic acid (δ-aminolevulinic acid, ALA) are assembled into the tetrapyrrole core structure (Ge et al. 2010).

The synthesis of ALA in animals, fungi and some bacteria was a condensation of glycine and succinyl-coenzyme A by ALA synthase (Tanaka and Tanaka 2007). However, in plants, algae and some photosynthetic bacteria, ALA is usually derived from the glutamate, which is called the five-carbon pathway (Grimm et al. 1991). In higher plants, ALA is the universal precursor of all tetrapyrroles, and its synthesis process is the rate-limiting step of tetrapyrroles biosynthesis (Lytovchenko et al. 2011). ALA was formed by transferance of the amino group of glutamate 1-semialdehyde (GSA) from C2 to C1, which was catalyzed by glutamate 1-semialdehyde 2,1-aminomutase (GSA aminotransferase, GSAM) (Grimm et al. 1991).

GSAM belongs to class-III pyridoxal-phosphate-dependent aminotransferase family (https://www.uniprot.org/uniprot/Q6YZE2), which was widely found in photosynthetic algae and plants (Song et al. 2016). In higher plants, the GSAM protein was first purified from the de-etiolated barley (*Hordeum vulgare*) seedings (Grimm et al. 1989), and *GSAM* genes have been successively identified from many species, such as soybean (*Glycine max*, Sangwan and O'Brian 1993), Arabidopsis (Song et al. 2016), tobacco (*Nicotiana tabacum*, Höfgen et al. 1994; PöRs et al. 2001), tomato (*Lycopersicon esculentum*, Polking et al. 1995; Lytovchenko et al. 2011), and *Brassica napus* (Tsang et al. 2003a). However, no mutant of *GSAM* has been identified in monocotyledonous plants. On the other hand, it was found that the GSAM could form a dimeric protein with itself (Grimm et al. 1989; Stetefeld et al. 2006; Ge et al. 2010), and different subunit states could show negative synergy when working (Hennig et al. 1997). Nonetheless, almost all researches on the dimeric structures of GSAM were conducted by x-ray crystallography, and no experiment on interaction of GSAM protein with itself has been reported so far.

In our research, we isolated a yellow leaf mutant, *ys53*, in rice (*Oryza sativa*). The mutant showed yellow phenotype throughout the whole growth period. Its photosynthetic pigment contents were decreased, and the chloroplasts showed obvious developmental impairment. In consequence, its major agronomic traits were significantly affected. Map-based cloning, intermediate product detection and complementation assays suggested that a single nucleotide mutation in *OsGSAM* (*LOC_Os08g41990*) is the reason for the mutant phenotype. *OsGSAM* is mainly expressed in green tissues and its encoded protein is localized to chloroplast. In addition, the yeast two-hybrid experiment showed that OsGSAM protein could interact with itself mainly by means of the two specific regions of amino acid residues at its N- and C-terminals, respectively.

## Results

### Isolation and Characterization of the *ys53* Mutant

**Fig. 1:**
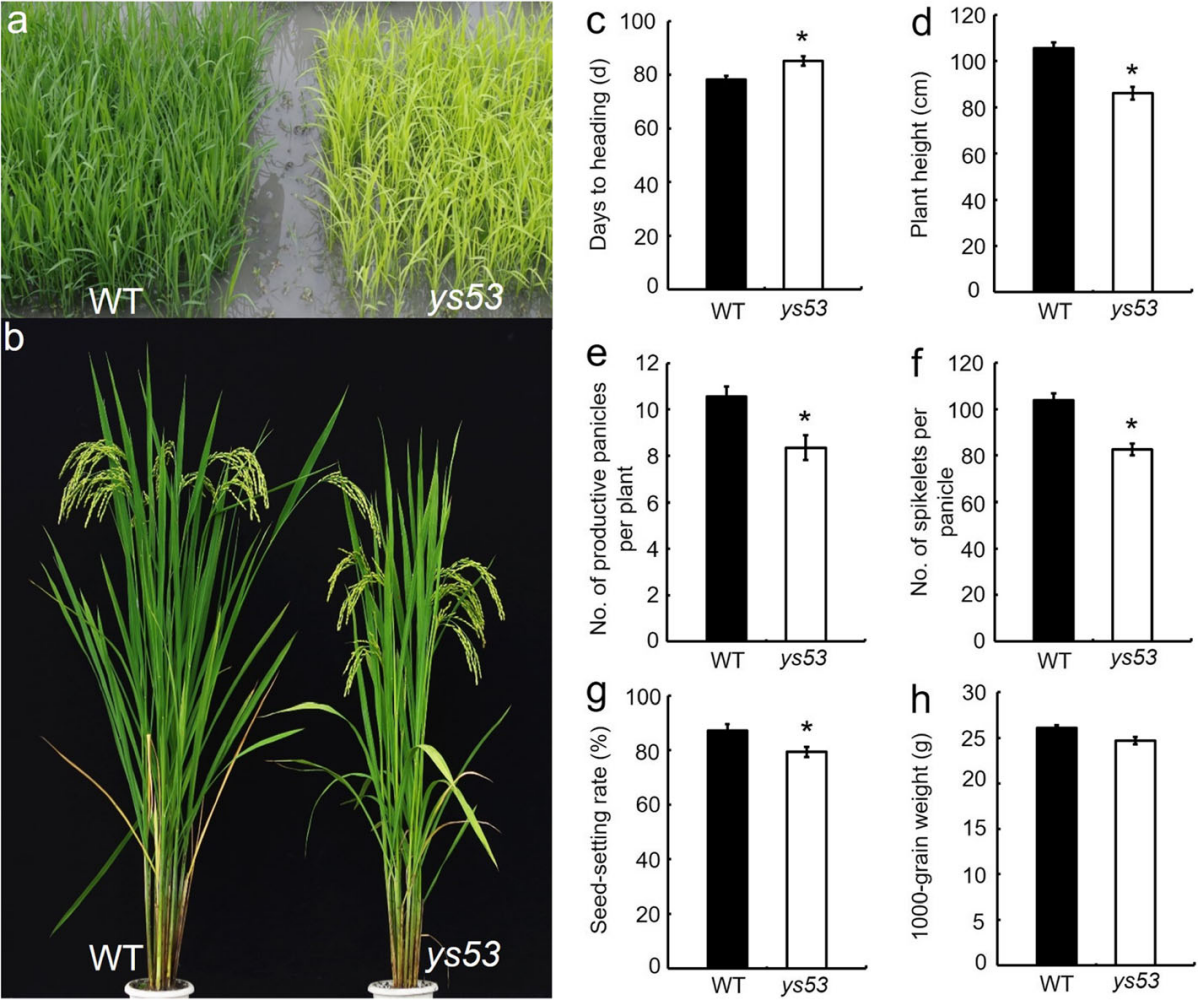
Comparison of plant phenotypes and major agronomical traits of the *ys53* mutant and its wild-type parent Nipponbare (WT). **a** Plant Phenotype at seedling stage. **b** Plant Phenotype at grain-filling stage. **c** Days to heading. **d** Plant height. **e** No. of productive panicles per plant. **f** No. of spikelets per panicle. **g** Seed-setting rate. **h** 1000-grain weight. Bars represent standard deviations (SDs) of three independent measurements. * signify statistically significant differences compared to the wild type at *P* < 0.05

The *ys53* mutant was obtained from *japonica* rice variety Nipponbare by ethyl methanesulfonate (EMS) mutagenesis. It exhibited a yellow leaf phenotype throughout the whole growth period, and grew at a slow rate (Fig. 1a, b), which caused its heading period to be delayed by 7 days (Fig. 1c). At maturity, except 1000-grain weight, major agronomic traits of *ys53* were significantly affected. For instance, its plant height, number of productive panicles per plant, number of spikelets per panicle and seed-setting rate were significantly reduced by 18.5%, 21.0%, 20.4% and 8.9%, respectively, compared with those of the wild type (Fig. 1d-h). In addition, because plant height of *ys53* was significantly lower than that of the wild type, we measured its internode and panicle length. The results showed that each internode of *ys53* was shortened proportionally, but its panicle length was not changed significantly (Additional file 2: Supplementary Fig. S1).

**Fig. 2:**
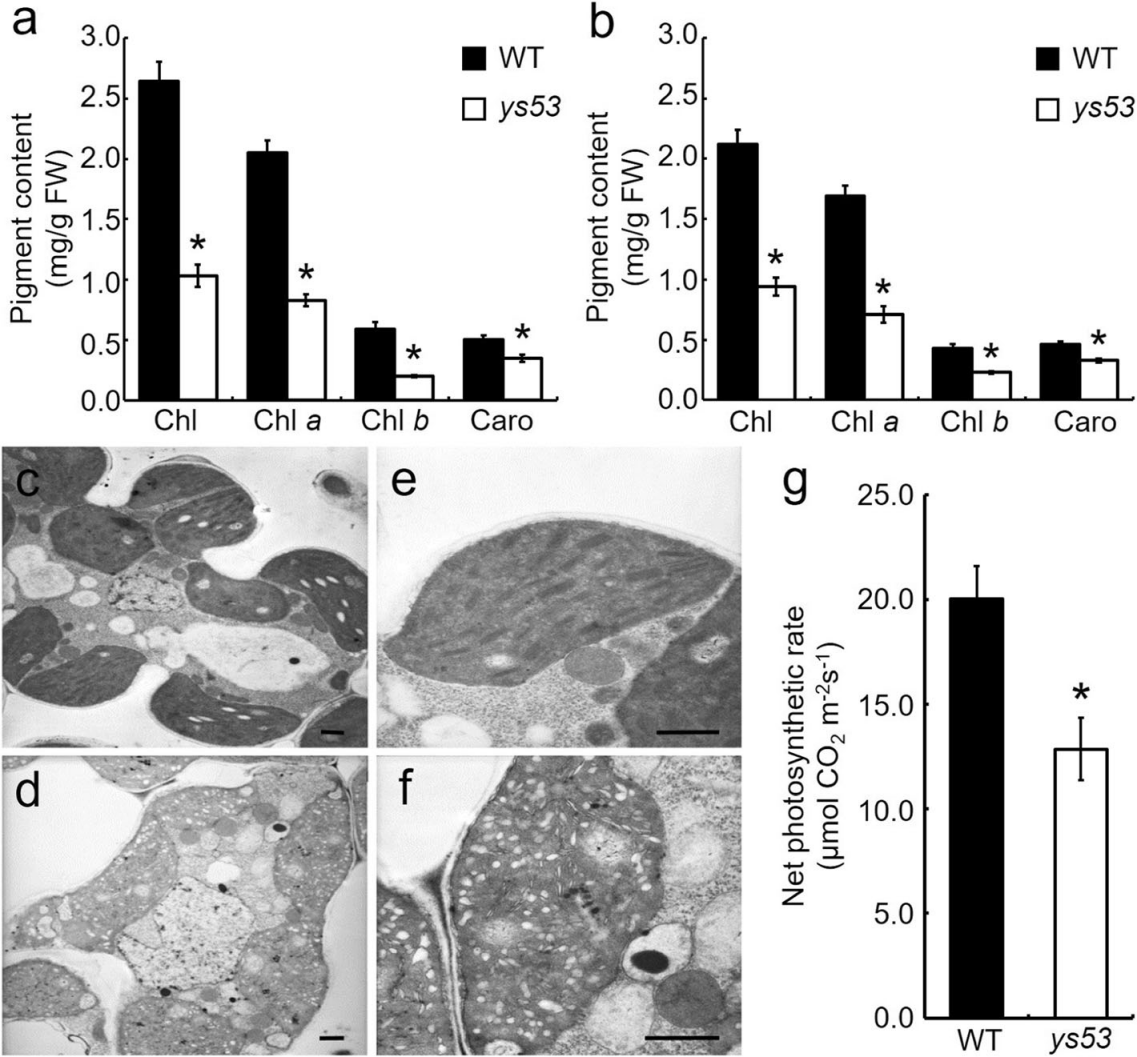
Physiological characteristics of the *ys53* mutant and its wild type. **a** and **b** Contents of photosynthetic pigments in leaves at seedling stage and booting stage, respectively. **c** and **d** Mesophyll cells of the wild type and *ys53*, respectively. **e** and **f** Chloroplasts of the wild type and *ys53*, respectively. **g** Net photosynthetic rate at grain-filling stages. Data are means ± SD (*n* = 3). Error bars represent (SDs) standard deviations of three independent experiments. * indicate statistically significant differences compared to the wild type at *P* < 0.05. Chl: chlorophyll. Caro: carotenoids. Scale bar = 1 μm

In order to further clarify the yellow phenotype of *ys53*, we determined photosynthetic pigment contents of the mutant and its wild type at different stages. At the seedling stage, contents of total chlorophyll (Chl), Chl *a*, Chl *b* and carotenoids (Caro) in leaves of *ys53* were significantly reduced by 61.0%, 59.5%, 66.1%, and 30.0% respectively, compared with those in the wild-type leaves (Fig. 2a). At the booting stage, total Chl, Chl *a*, Chl *b* and Caro contents in the mutant leaves were also significantly decreased by 55.7%, 58.0%, 46.5% and 28.3%, respectively (Fig. 2b). These data suggested that the yellow leaf phenotype of *ys53* was due to the defect in photosynthetic pigments.

To explore effect of the decreased pigment levels on the chloroplast structure of *ys53*, the ultrastructure of chloroplasts was observed under a transmission electron microscope. In wild type, chloroplasts had intact thylakoid and granum structure, with dense and ordered grana stacks. In contrast, *ys53* mutant chloroplasts had no intact thylakoid and granum structure, and exhibited obvious vacuolation (Fig. 2c-f). The result indicated that the development of chloroplast was suppressed in the *ys53* mutant.

Next, we investigated the photosynthetic capacity of *ys53*. As shown in Fig. 2g, net photosynthetic rate of the *ys53* flag leaves at the grain-filling stage was significantly lower than that of the wild type, suggesting that decreased level of pigments and suppressed development of chloroplasts significantly affected the photosynthetic capacity of the *ys53* mutant.

In addition, to explore whether the yellow leaf phenotype of *ys53* was affected by temperature, we treated *ys53* and wild-type seedlings grown in the growth chamber using two different temperature conditions (constant 23 °C and 30 °C), respectively. As a result, the *ys53* mutant grown under different temperature conditions exhibited similar leaf-color phenotype and decrease degree of photosynthetic pigment contents (Additional file 2: Supplementary Fig. S2), which indicated that the yellow phenotype of *ys53* was independent upon temperature.

### Map-Based Cloning of the *ys53* Mutant Gene

**Fig. 3:**
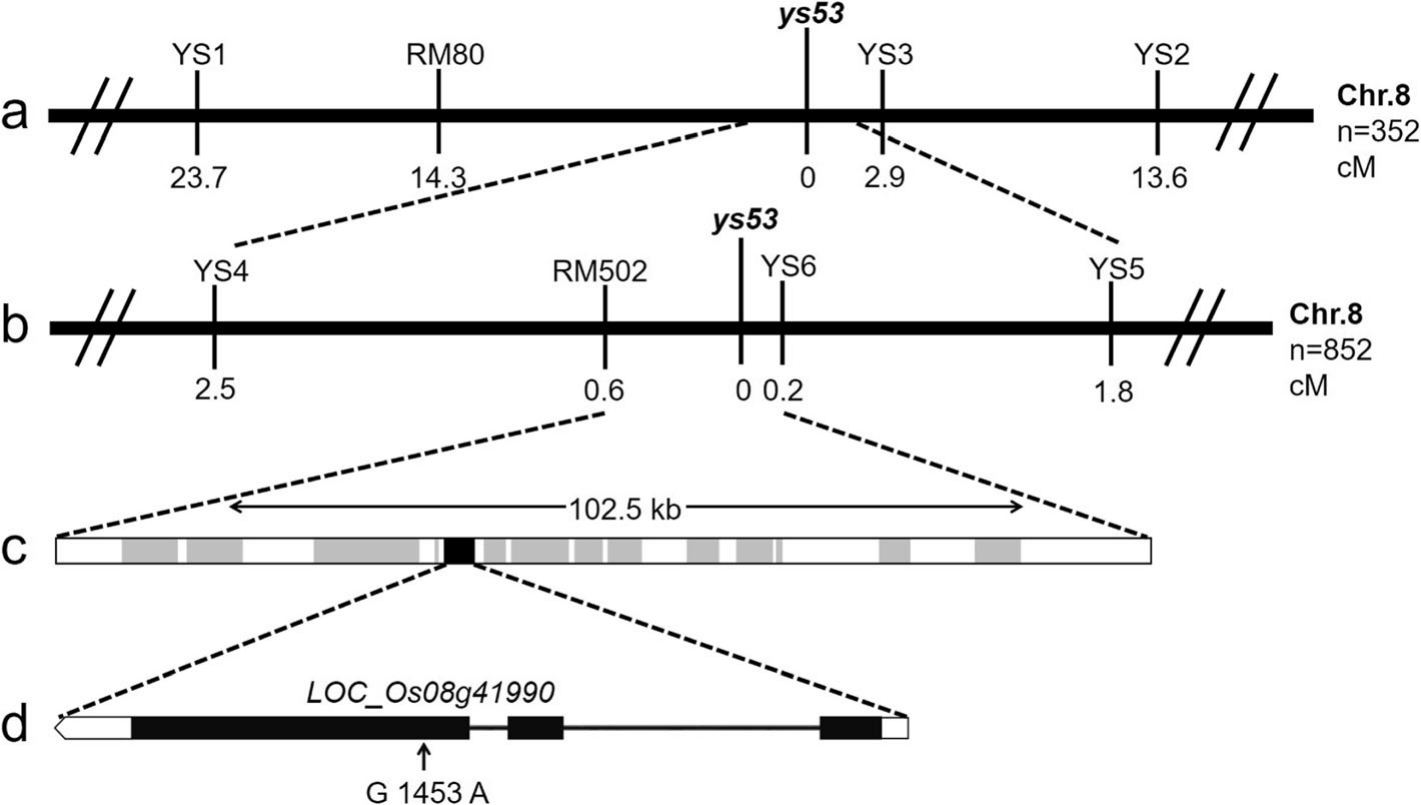
Map-based cloning of the *ys53* locus. **a** The *ys53* locus was mapped to the long arm of rice chromosome 8 using 352 F_2_ yellow-leaf individuals. **b** This locus was further mapped to a 102.5-kb region between SSR marker RM502 and InDel marker YS6 using 852 F_2_ yellow-leaf individuals. **c** This region contains 14 putative genes. **d** Candidate gene *LOC_Os08g41990* comprises three exons and two introns, in which a single nucleotide G-to-A substitution occurred at position 1453 of its coding region in the mutant

For genetic analysis of the mutant, *ys53* was crossed with normal *indica* restorer line Minghui 63. The resulting F_1_ plants exhibited a normal green phenotype, and leaf-color phenotypes of the F_2_ population segregated with a ratio of 3:1 (χ^2^ < χ^2^_0.05_ = 3.84, *P* > 0.05, Additional file 1: Supplementary Table S1). The result suggested that the yellow leaf phenotype of the mutant was controlled by a single recessive nuclear gene. Then, SSR and InDel markers and the (*ys53*/Minghui 63) F_2_ population were used to map the mutant gene. The initial mapping result with 352 F_2_ yellow-leaf individuals showed that the target gene *ys53* was linked to the SSR marker RM80 located on the long arm of Chromosome 8 (Fig. 3a). After that, the 852 F_2_ yellow-leaf plants and the seven InDel and SSR markers with polymorphism between the two parents were used to fine map the *ys53* locus (Table 1). Finally, the target locus was narrowed to 102.5-kb region between SSR marker RM502 and InDel marker YS6, at genetic distance of 0.6 cM and 0.2 cM, respectively (Fig. 3b).

**Table 1:** Polymorphic InDel markers developed in this study

Marker	Forward primer (5′-3′)	Reverse primer (5′-3′)
YS1	AGTATCATTTCGGTTTGGT	CATTCGTGGTTGTATTGG
YS2	TAGCCGATCCGAACTTGC	GTCTGACCTGCCGACCAA
YS3	TGGTGATGTCTAGCTCAATC	TGATAGGTGGATAGCAGT
YS4	ATGCCCGTGCTTCGGTAC	CTGTCATTGGAGACTTCG
YS5	GGACCTTTACCCTGGCGC	GACGAGCTGGCACACATTC
YS6	GCTAGATTGCTCTCTGTGC	CTTGCAATTGACATTACC

Within the above-mentioned 102.5-kb region, 14 putative genes are annotated by Rice Genome Annotation Project (http://rice.plantbiology.msu.edu) (Fig. 3c, Additional file 1: Supplementary Table S2). Among these genes, only one gene (*LOC_Os08g41990*) contains a chloroplast transit peptide according to the analysis result using ChloroP and TargetP (http://www.cbs.dtu.dk/services/ChloroP/; http://www.cbs.dtu.dk/services/TargetP/). Meanwhile, 9 genes locating on the middle of this region were first amplified from *ys53* genomic DNA and sequenced one by one (Additional file 1: Supplementary Table S3), and only one mutation was found and furthermore, occurred in the *LOC_Os08g41990* gene in *ys53* mutant. Then, we sequenced cDNA of this gene from *ys53* mutant and its wild-type parent Nipponbare using reverse transcription (RT)-PCR, and confirmed the point mutation mentioned above. This gene encodes an aminotransferase with 78.9% and 75.7% identities to Arabidopsis GSA1 and GSA2 respectively, which participate in tetrapyrrole biosynthetic pathway in higher plants. In the *ys53* mutant, a single nucleotide G to A substitution occurred at position 1453 of *LOC_Os08g41990* genome sequence (corresponding to position 511 of its cDNA) (Fig. 3d), causing an amino acid change in its encoded protein. Therefore, *LOC_Os08g41990* was considered as the candidate gene of *ys53* mutant, and designated tentatively as *OsGSAM*.

Database searching showed that the *OsGSAM* gene is a single copy gene in rice genome. Sequencing revealed that this gene contains three exons and two introns. The full length of its genomic sequence is 2379 bp and the cDNA length is 1437 bp. The protein encoded by *OsGSAM* consists of 478 amino acids and is predicted to have approximately 50 kDa molecular weight. The OsGSAM protein contains two domains, a chloroplast transit peptide of 40 amino acid residues at N-terminus and an aminotransferase class-III of 397 amino acid residues at C-terminus (Additional file 2: Supplementary Fig. S3) (http://smart.embl-heidelberg.de/). Meanwhile, it has 20 α-helixes and 21 β-sheets, and the mutation site in *ys53* mutant was located on a conserved site of the 8th α-helix (Additional file 2: Supplementary Fig. S3) (https://swissmodel.expasy.org/interactive).

According to the multiple sequence alignment of OsGSAM and its homologues in different species, OsGSAM has a high similarity to its homologues in monocotyledonous plants, maize (*Zea mays*), and barley (*Hordeum vulgare*) and dicotyledonous plants, *Arabidopsis thaliana* (GSA2, GSA1), tobaco (*Nicotiana attenuate*) and cucumber (*Cucumis satvus*), with 92.2%, 90.7%, 78.9%, 75.7%, 78.3%, 76.6% respectively. OsGSAM also has 76.6% and 73.4% similarity to its homologues in single-celled algaes *Chlamydomonas reinhardtii* and *Synechococcus*, respectively. In addition, phylogenetic analysis showed that OsGSAM is more closely related to GSAM homologues from maize and barley than those from other species (Additional file 2: Supplementary Fig. S4).

### GSA Accumulation and ALA-Synthesis Ability in *ys53* Mutant

**Fig. 4:**
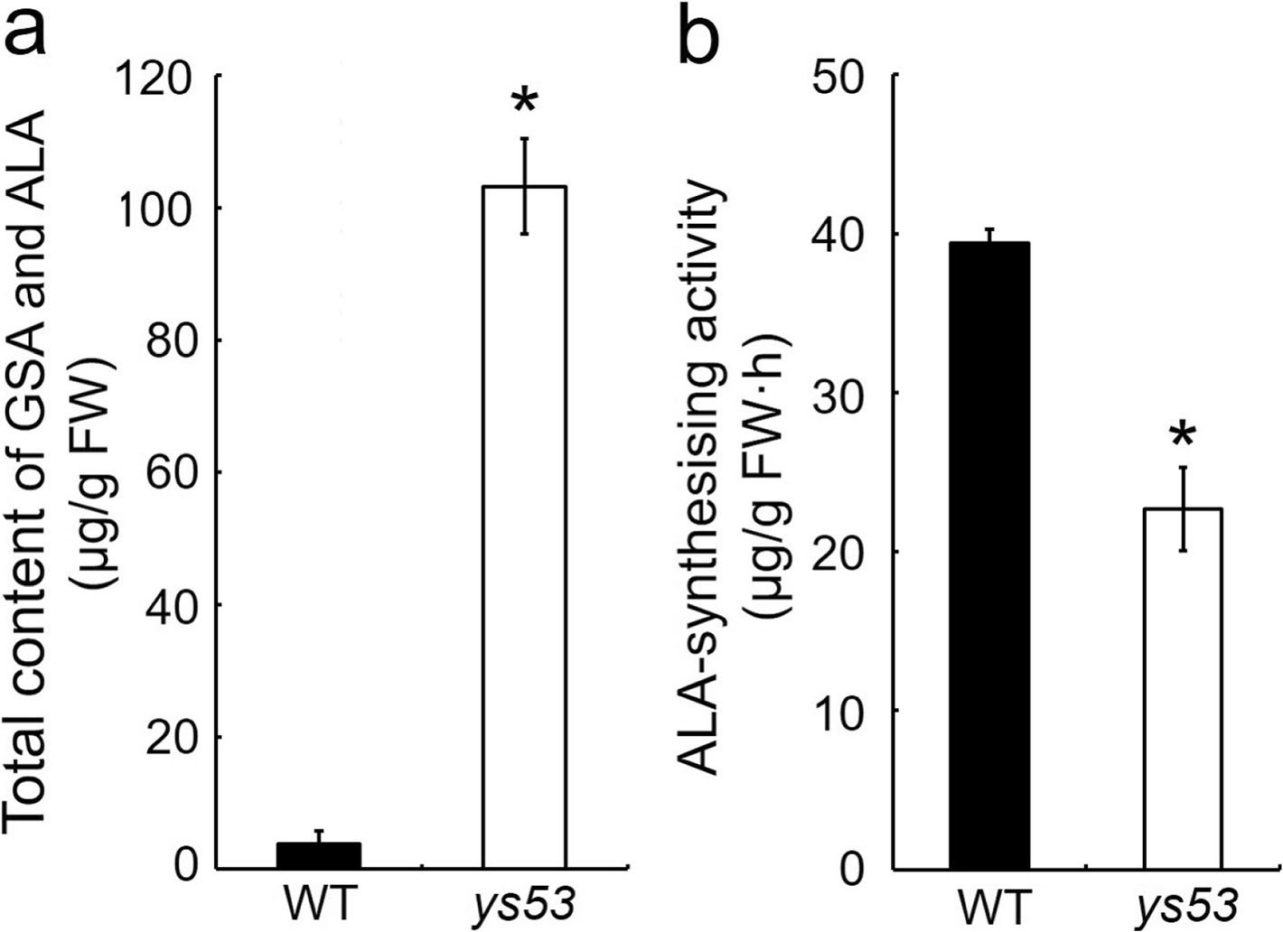
Determination of GSA and ALA content and ALA-synthesizing activity in *ys53* and its wild type. **a** Total content of GSA and ALA in rice leaves at seedling stage. Leaves were harvested from normal growing seedlings in the paddy field on sunny afternoons, quick freezed in liquid nitrogen and used for determination of total content of GSA and ALA. **b** ALA-synthesizing activity of rice seedlings. After 1 week of dark culture, the yellow seedlings of *ys53* and its wild type were cultured under light condition (100 μmol m^− 2^ s^− 1^) for 7 h in LA (levulinic acid) solution, and then the measurement of GSA and ALA was performed. Error bars represent the SDs of three independent experiments. * indicates statistically significant difference between *ys53* and its wild type at *P* < 0.05

GSAM converts GSA to ALA by transferance of the amino group of GSA from C2 to C1 in higher plants (Tanaka and Tanaka 2007). If the *ys53* mutant indeed has a defective GSAM, its ALA-synthesising capacity should be decreased, resulting in massive accumulation of GSA in the mutant plants. So we should first examine GSA content in *ys53*. However, GSA is an isomer of its product ALA, and both GSA and ALA can react with acetylacetone to form a pyrrole that further reacts with Ehrlich’s reagent to form a colored compound. Then, we compared total content of GSA and ALA between *ys53* and its wild type using the method of Mauzerall and Granick (1955) with slight optimization. The results showed that total content of GSA and ALA in *ys53* mutant reached to 103.3 μg/g·FW, but that in the wild type was only 3.9 μg/g·FW under natural condition (Fig. 4a). Generally, GSA is stable in dilute solution at acidic pH (such as the acidic condition in the present study) (Houen et al. 1983; Kannangara and Schouboe 1985). On the other hand, ALA is undetectable because it is very quickly converted to porphobilinogen by ALA dehydratase in normal plants (Richter et al. 2010). So the overwhelming majority of total content of GSA and ALA should actually be GSA content in *ys53* mutant. Therefore, the data suggested that GSA massively accumulated in the mutant.

On the other hand, LA (levulinic acid) has a similar molecular structure to ALA and can compete with ALA to bind to ALA dehydratase, so excess LA can block the conversion of ALA to porphobilinogen. Therefore, we cultivated yellow seedlings of *ys53* and its wild type under light and excess LA conditions to compare ALA-synthesis capacity of them. As shown in Fig. 4b, the ALA-synthesis capacity of *ys53* was 22.6 μg/g·FW·h, while that of wild type was 39.4 μg/g·FW·h, which showed that ALA-synthesis ability of the mutant was significantly reduced by 42.6% compared with that of the wild type.

Taken together, we concluded that GSAM protein should be defective in the *ys53* mutant.

### Complementation of the *ys53* Mutant

**Fig. 5:**
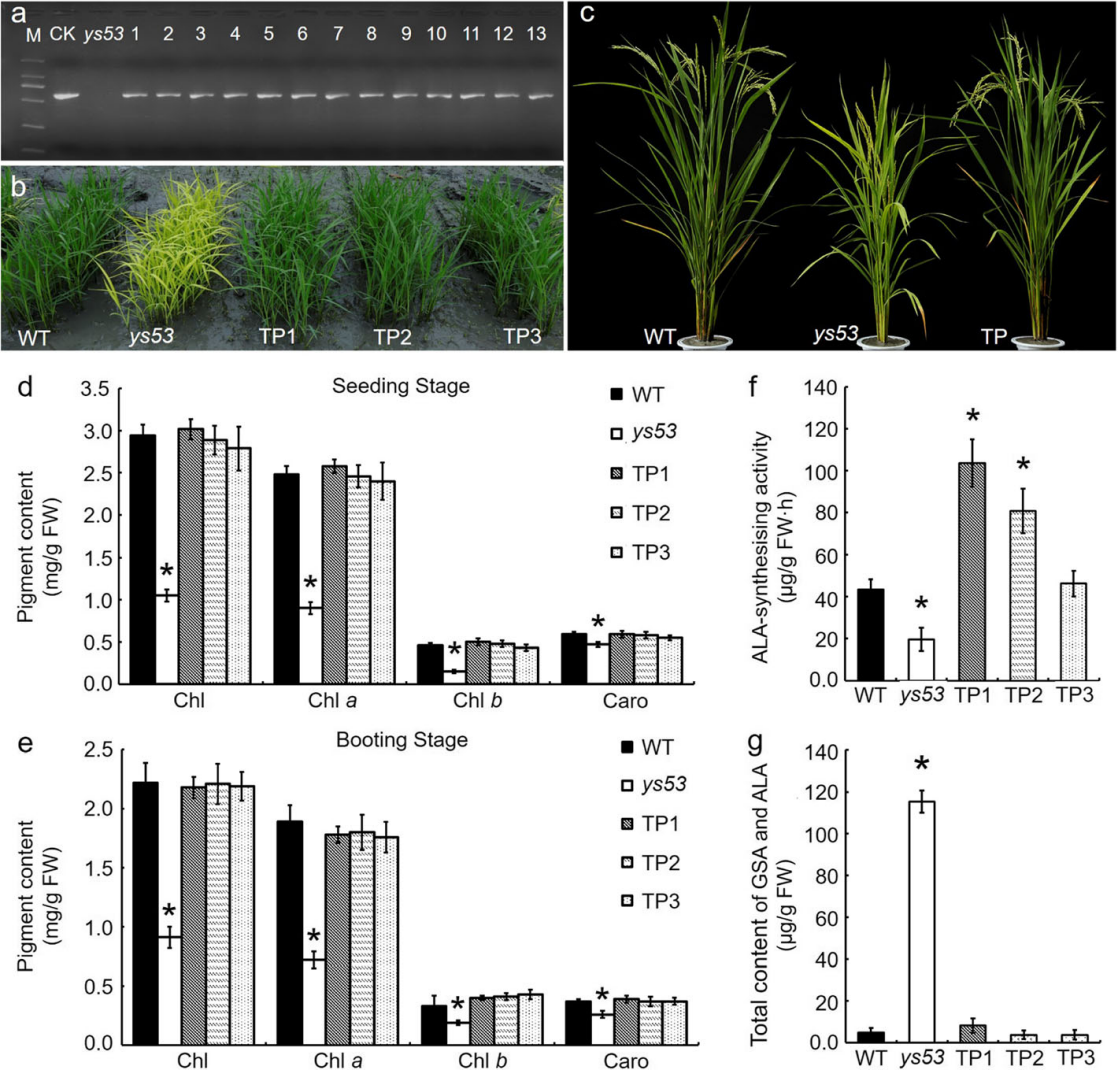
Complementation of the *ys53* mutant by *OsGSAM* gene. **a** Identification of transgenic lines by PCR. M, DL-2000 Marker; CK, pC2300-*OsGSA* plasmid (PCR positive control); *ys53*, *ys53* mutant (PCR negative control); 1–13, positive transgenic plants. **b** Phenotypes of *ys53* mutant, the wild type (WT), and the positive transgenic plants (TPs) at seedling stage. **c** Phenotypes of *ys53*, WT, and TPs at grain filling stage. **d** Photosynthetic pigment contents (in mg g fresh weight^− 1^) of *ys53*, WT, and TPs in leaves of four-week-old seedlings. **e** Photosynthetic pigment contents (in mg g fresh weight^− 1^) in leaves of *ys53*, WT, and TPs at booting stage. **f** and **g** ALA-synthesising activity of rice seedlings, and total content of GSA and ALA in rice leaves at seedling stage, respectively, which were detected according to the methods as Fig. 4. Data are mean ± SD (*n* = 3). Error bars represent SDs of three independent experiments. * indicate statistically significant differences compared with WT at *P* < 0.05

In order to further confirm that the yellow leaf phenotype of *ys53* was caused by the mutation in the *OsGSAM* gene, we constructed complementary vector and carried out transgenic experiment. First, the full-length cDNA of the wild-type *OsGSAM* gene was amplified, and inserted into the pCAMBIA2300 vector containing the *Actin 1* promoter. Then, the fusion vector pCAMBIA2300-*OsGSAM* was introduced into the *ys53* mutant by *Agrobacterium*-mediated transformation. As a result, a total of 13 positive transgenic lines were obtained through identification with PCR (Fig. 5a). These transgenic lines all showed normal leaf-color phenotype as the wild type (Fig. 5b, c). Meanwhile, we measured pigment contents in the positive transgenic lines at seedling stage and booting stage, respectively. As shown in Fig. 5d and e, pigment contents of transgenic lines TP-1, TP-2 and TP-3 were not significantly different from those of the wild type. In addition, we tested GSA accumulation and ALA-synthesis ability of TP-1, TP-2 and TP-3. The results showed that the three positive transgenic lines had significant increase of ALA-synthesis ability compared with *ys53* mutant (Fig. 5f), and they had no obvious accumulation of GSA as the wild type (Fig. 5g). In a word, these data suggested that the *OsGSAM* gene rescued the yellow leaf phenotype of *ys53*, confirming that the mutant phenotype was a consequent of the single-base mutation of *OsGSAM* gene.

### Subcellular Localization of OsGSAM Protein

**Fig. 6:**
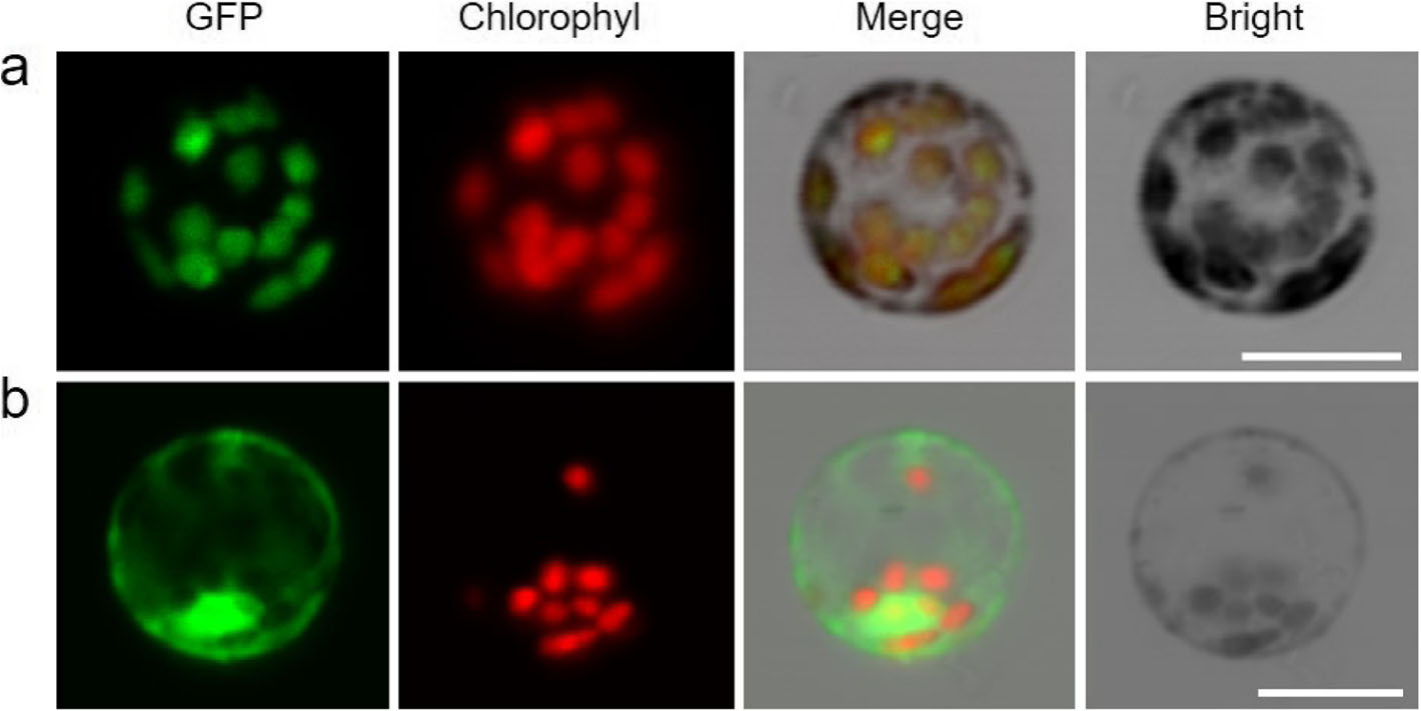
Subcellular localization of OsGSAM protein. **a** GFP signals of the OsGSAM-eGFP fusion protein. **b** Empty vector eGFP without a specific targeting sequence. Green fluorescence shows GFP, red fluorescence indicates chloroplast autofluorescence, yellow fluorescence indicates images with the two types of fluorescence merged, and bright field images shows rice protoplasts. Fluorescence signals were visualized using a laser-scanning confocal microscopy. Bars = 10 μm

OsGSAM was predicted to be located in chloroplast because it contains a chloroplast transit peptide with 30 amino acid residues at its N-terminus. To verify this prediction, we made pCAMBIA2300-35S-*OsGSAM*-*GFP* vector, a construct expressing the OsGSAM-GFP (green fluorescent protein) fusion protein, and transformed rice protoplast using the resulting construct and the empty vector pCAMBIA2300-35S-GFP (as control) respectively. Then, the transformed protoplasts were observed by laser-scanning confocal microscopy. As expected, the green fluorescence of OsGSAM-GFP fusion protein exactly overlapped with the red autofluorescence of chlorophyll in chloroplasts (Fig. 6a). Meanwhile, the green fluorescence of empty vector was diffusely distributed in the protoplast (Fig. 6b). This result suggested that OsGSAM is indeed located in chloroplast.

### Expression Pattern of *OsGSAM* Gene

**Fig. 7:**
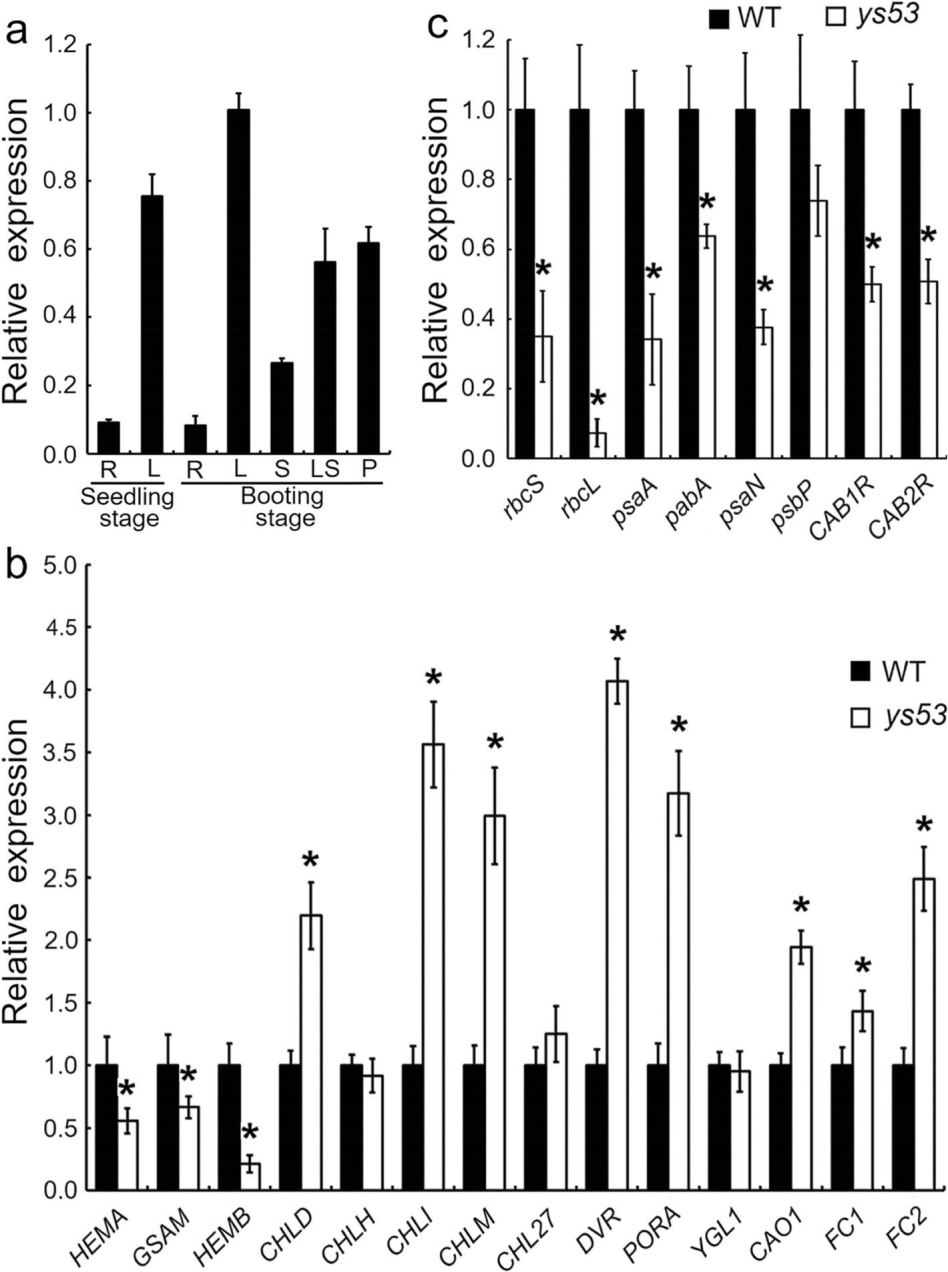
Expression analyses of related genes by qRT-PCR. **a** Expression pattern of *OsGSAM* gene. The total RNA was extracted from different tissues, including root (R), leaf blade (L), stem (S), leaf sheath (LS) and young panicle (P), of the wild type at seedling stage and booting stage, respectively. Rice *actin 1* gene was used as an internal control. Error bars represent the SDs of three independent experiments. **b** and **c** Comparison of expression levels of the genes associated with tetrapyrrole biosynthesis and photosynthesis, respectively, between *ys53* and its wild at seedling stage. The total RNA was extracted from 4-week-old seedlings. The relative mRNA amount of each gene was normalized to *Actin 1*. The expression level of each gene in the wild type was set to 1.0, and those in *ys53* mutant were calculated accordingly. Error bars represent the SDs of three independent experiments. Asterisks indicate statistically significant differences compared with the wild type at *P* < 0.05

In order to explore the expression pattern of *OsGSAM*, its transcriptional levels in different tissues of the wild type were measured by qRT-PCR at the seedling stage and the booting stage, respectively. The data showed that *OsGSAM* was expressed in wide range of tissues, including roots, stems, leaf sheaths, leaf blades and young panicles. Nonetheless, *OsGSAM* was differentially expressed in different tissues. Specifically, leaf blades had the highest expression, followed by young panicles and leaf sheaths, while stems and roots had lower levels of expression (Fig. 7a).

### Expression Analysis of the Genes for Tetrapyrrole Biosynthesis and Photosynthesis

Due to the contents of photosynthetic pigments and their precursor ALA in the *ys53* mutant were significantly reduced, we examined transcription levels of the 14 genes involved in tetrapyrrole biosynthesis by qRT-PCR at seedling stage. Among them, three genes encode the enzymes for the common steps of tetrapyrrole biosynthetic pathway, including glutamyl-tRNA reductase (GluTR, *HEMA*) (Apitz et al. 2016), *GSAM*, and 5-aminolevulinate dehydratase (*HEMB*) (Tang et al. 2012); nine genes encode the enzymes for chlorophyll biosynthesis, including magnesium chelatase D subunit (*CHLD*), chelatase H subunit (*CHLH*), magnesium chelatase I subunit (*CHLI*), magnesium chelatase M subunit (*CHLM*), Mg-protoporphyrin IX monomethylester cyclase (*CHL27*), 3,8-divinyl protochlorophyllide *a* 8-vinyl reductase (*DVR*), protochlorophyllide oxidoreductase A (*PORA*), Chl synthase (*YGL1*) and chlorophyllide *a* oxygenase (*CAO1*) (Inagaki et al. 2015; Li et al. 2019; Kong et al. 2016; Wang et al. 2010; Sakuraba et al. 2013; Wu et al. 2007; Lee et al. 2005); and two genes encode ferrochelatase1 and 2 (*FC1* and *FC2*) for the heme branch (Inagaki et al. 2015). As shown in Fig. 7b, all the three genes (*HEMA, GSAM* and *HEMB*) for the common steps were significantly down-regulated in the *ys53* mutant, compared with the wild type. In the 11 genes for chlorophyll and heme branches, only *CHLH*, *CHL27* and *YGL1* did not significantly change in expression level, and the other eight genes, including *CHLD*, *CHLI*, *CHLM*, *DVR*, *PORA*, *CAO1*, *FC1* and *FC2*, were all significantly up-regulated in the mutant.

Besides, the chloroplast development of *ys53* mutant was suppressed, and its net photosynthetic rate was significantly decreased, so we also performed qRT-PCR to explore expression levels of the eight genes associated with photosynthesis at seedling stage. The proteins encoded by these genes were as follows: rubisco small/large subunit (*rbcS* and *rbcL*), two reaction center polypeptides (*psaA* and *psbA*)), reaction center subunit of PSI (*psaN*), thylakoid luminal subunit of PSII (*psbP*), and light-harvesting Chl *a*/*b*-binding protein of PSII (*CAB1R* and *CAB2R*) (Li et al. 2015; Su et al. 2012; Amunts et al. 2010; Ifuku et al. 2008; Ma et al. 2017). The result showed that expression level of *psbP* did not significantly change, but those of the other seven genes were all significantly decreased in the mutant relative to those in the wild type (Fig. 7c).

The above data indicated that the mutation of *OsGSAM* not only affected the expressions of tetrapyrrole biosynthetic genes, but also influenced those of photosynthetic genes in rice.

### Protein Interaction of OsGSAM with Itself

GSAM protein was first found to exist as dimer in barley and *Synechococcus* (Grimm et al. 1989). To date, the dimers of GSAM were usually identified by x-ray crystallography in many species, such as *Escherichia coli*, *Synechococcus* and Arabidopsis (Ilag et al. 1991; Hennig et al. 1997; Zhao et al. 2014). Here we carried out a series of yeast two-hybrid experiment to explore whether rice GSAM exists in the same form of dimer and where the dimeric protein was formed by interaction.

**Fig. 8:**
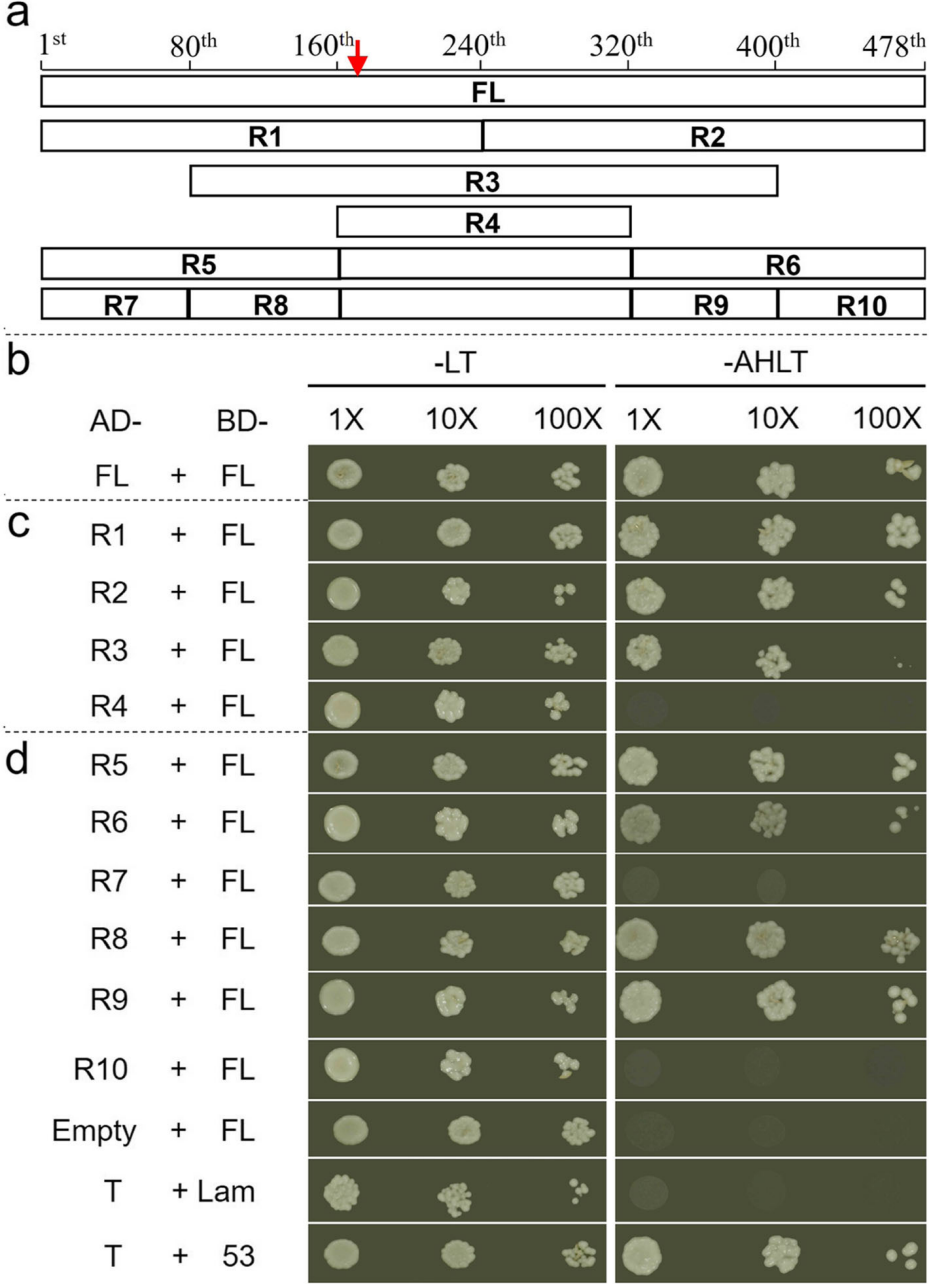
OsGSAM protein truncation experiment. **a** Schematic diagram of OsGSAM protein truncation. FL represents the full-length OsGSAM, and R1-R10 correspond to truncated fragments containing the 1st-240th, 241th–478th, 81th–400th, 161th–320th, 1st-160th, 321th–478th, 1st-80th, 81th–160th, 321th–400th and 401th–478th amino acid residues of OsGSAM, respectively. The red arrow indicates the substitution of threonine for alanine at the 171th amino acid residue of OsGSAM in the *ys53* mutant. **b** The yeast two-hybrid experiment of the full-length OsGSAM with itself. **c** Yeast two-hybrid experiments between the R1, R2, R3 and R4 fragments and the full-length GSAM. **d** Yeast two-hybrid experiments between the R5, R6, R7, R8, R9 and R10 fragments and the full-length GSAM. The FL was combined with empty pGADT7 vector as internal control. Interaction between pGADT7-T and pGBKT7-Lam was used as a negative control, and interaction between pGADT7-T and pGBKT7–53 was used as a positive control. Co-transformed yeast colonies were spotted on the selective SD medium minus Trp and Leu (−LT), the normally surviving monoclonal bacteria were selected and then dripped to the SD medium minus His, Leu, Trp and Ade to grow (−AHLT). 1X, 10X, and 100X represent the saturated culture solution of transformant diluted to concentrations of 1, 0.1 and 0.01, respectively

First, the full-length cDNA sequence of *OsGSAM* (FL-cDNA) was fused to GAL4 AD and GAL4 BD vectors respectively, and the resulting constructs were co-transformed into Y2H Gold yeast cells. The result showed that OsGSAM could interact with itself (Fig. 8b), which was consistent with the phenomenon of GSAM dimer in the previous reports.

Next, the FL-cDNA was divided into four different fragments encoding R1, R2, R3 and R4 peptide sequences, respectively (Fig. 8a). The four fragments were fused to GAL4 AD, and the resulting constructs were co-transferred with the GAL4 BD construct containing FL-cDNA into Y2H GOLD cells, respectively. The data showed that R1, R2 and R3 could interact with the full-length GSAM protein, but R4 could not (Fig. 8c), implying that OsGSAM might contain two different interaction regions at its N- and C-ends, respectively.

After that, the FL-cDNA was again divided into six different fragments encoding R5, R6, R7, R8, R9 and R10 peptide sequences, respectively (Fig. 8a). The six fragments were fused to GAL4 AD, and the resulting constructs were co-transferred with the GAL4 BD construct containing FL-cDNA into Y2H GOLD cells, respectively. As shown in Fig. 8d, R5, R6, R8 and R9 could interact with the full-length GSAM protein, but R7 and R10 could not. These results indicated that the interaction of OsGSAM with itself could largely depend on the two specific regions R8 and R9 which contain the 81th–160th and the 321th–400th amino acid residues at the N- and C-terminals of OsGSAM, respectively.

## Discussion

GSAM protein was first purified from barley and cyanobacteria *Synechococcus* using affinity chromatography and polyacrylamide gel electrophoresis under non-denaturing conditions (Grimm et al. 1989). So far, *GSAM* genes have only been identified via homology cloning strategy or RNA interference in many plant species, such as soybean (Sangwan and O'Brian 1993), tobacco (Höfgen et al. 1994; PöRs et al. 2001), tomato (Polking et al. 1995; Lytovchenko et al. 2011), and *Brassica napus* (Tsang et al. 2003a). However, no mutant of *GSAM* has been reported in monocotyledonous plants. In this study, a rice yellow-leaf mutant *ys53* was isolated, which showed a yellow phenotype, reduced contents of pigments, and impaired development of chloroplasts. Then, we cloned the *OsGSAM* gene by means of the *ys53* mutant and map-based cloning approach, and found that a single nucleotide substitution occurred in the mutated gene of *ys53*, causing an amino acid change in its encoded protein. Furthermore, the yellow leaf phenotype of the mutant could be rescued by introducing the wild-type *OsGSAM* (Fig. 5). Therefore, we confirmed that the point mutation of *OsGSAM* was the cause of the yellow phenotype of this mutant, and successfully identified a *GSAM* gene in monocotyledonous plants through map-based cloning.

Up to now, a few of *GSAM* RNAi plants have been reported in higher plants. Among them, the *GSAM* RNAi plants of tabaco showed different degrees of chlorophyll reduction and variegation patterns (Höfgen et al. 1994). *Brassica napus* plants expressing a *GSAM* antisense gene directed by a *Brassica* napin promoter exhibited yellow cotyledons and a range of chlorophyll reduction in the seeds, but the seed growth and development were not affected (Tsang et al. 2003b). The tomato plants expressing antisense *GSAM* gene directed by TFM5 green fruit-specific promoter displayed lower chlorophyll levels and photosynthetic activity, but almost no differences in fruit size, weight, or ripening capacity (Lytovchenko et al. 2011). In this study, a single-base mutation of *OsGSAM* led to an amino acid substitution of the encoded protein, and finally caused yellow leaf phenotype throughout the whole growth period in *ys53* mutant (Fig. 1a, b). Meanwhile, its pigment contents were reduced (Fig. 2a, b), chloroplast development was suppressed (Fig. 2c-f), and the net photosynthetic rate declined (Fig. 2g). In consequence, its plant height, number of productive panicles per plant, number of grains per panicle and seed setting rate were significantly reduced, but its grain weight was almost unchanged (Fig. 1d-g). The above phenotypes of *ys53* were not completely consistent with the phenotypes of RNAi lines of tabaco, *Brassica napus* and tomato as previously reports, which could be due to the differences between a point mutation and RNA interferences and among the different plant species.

Structure investigations revealed that the *Synechococcus* GSAM protein contains four clusters of invariant residues, including (i) substrate binding residues (Ser-29–Arg-32 and Glu-406), (ii) interface helix residues (Ser-122–Ala-126, Arg-132, and Arg-135), (iii) residues within the gating loop (Tyr-150–His-153, Leu-158, Ser-163, and Thr-167), and (iv) residues fixing the phosphate group of cofactor of the opposite subunit (Tyr-301*–Thr-305*) (Stetefeld et al. 2006). Each of the four clusters is conservative in different species, and involved in substrate binding, cofactor fixation, formation of the interface helix, or recruiting of the gating loop (Stetefeld et al. 2006). In the present study, the mutation site (the 171th amino acid residue) of *ys53* is located on cluster (ii) of invariant residues (the interface helix residues), corresponding to Ala-126 of *Synechococcus* GSAM (Additional file 2: Supplementary Fig. S3). Therefore, we speculated that the mutation of Ala-171 to Thr could affect biological function of OsGSAM, resulting in significant reduction of ALA-synthesising capacity and the yellow leaf phenotype of the *ys53* mutant.

GSAM protein was first found to exist as a dimer in barley and *Synechococcus* (Grimm et al. 1989). However, almost all studies on the dimers and structures of GSAM were performed by x-ray crystallography, for example, in *E. coli* (Ilag et al. 1991), cyanobacteria *Synechococcus* (Hennig et al. 1997; Stetefeld et al. 2006) and Arabidopsis (Zhao et al. 2014). So far, no other form of experimental evidence confirmed the dimeric phenomenon of this protein, and which region drives the formation of this dimer has not been reported. In the present study, we found that OsGSAM protein can interact with itself through yeast two-hybrid experiment (Fig. 8b), which is consistent with the phenomenon of GSAM dimer in the previous reports (Grimm 1990; Hennig et al. 1997; Contestabile et al. 2000; Stetefeld et al. 2006). Meanwhile, our further research indicated that the interaction of OsGSAM with itself could largely depend on the two specific regions R8 and R9 containing the 81th–160th and the 321th–400th amino acid residues at the N- and C-terminals of OsGSAM, respectively (Fig. 8c, d), which might be one aspect of the foundation leading to the formation of GSAM dimer.

## Conclusions

*YS53* encodes glutamate-1-semialdehyde 2,1-aminomutase (GSAM) participating in tetrapyrrole biosynthesis. In *ys53* mutant, a single nucleotide substitution of *YS53* gene causes massive accumulation of glutamate 1-semialdehyde, leading to yellow leaf phenotype in rice. In addition, yeast two-hybrid experiment suggested that rice GSAM protein could interact with itself mainly by means of the two specific regions of amino acid residues at its N- and C-terminals, respectively.

## Materials and Methods

### Plant Materials and Growth Conditions

A yellow leaf mutant, *ys53*, was obtained from *japonica* cultivar Nipponbare through ethyl methanesulfonate (EMS) mutagenesis. The mutant was crossed with normal *indica* restorer line Minghui 63 to construct the F_1_ and F_2_ populations for genetic analysis and gene mapping. Rice materials were grown in a paddy field under the local growing environment in Wenjiang District (latitude 30°42′N, longitude 103°50′E and altitude 539.3 m), Chengdu, Sichuan, China (Wang et al. 2010; Wang et al. 2014). In addition, to investigate effect of temperature on the phenotype of *ys53*, rice seedlings were grown in the growth chamber under 12 h of light (80–100 μmol m^− 2^ s^− 1^)/12 h of dark at constant 23 °C (low temperature) or 30 °C (high temperature) (Huang et al. 2017; Chen et al.^ 2013^).

### Measurement of Photosynthetic Pigments

Leaf samples were collected from *ys53* and its wild type at seedling stage and booting stage respectively. 0.2 g leaf was used to extract pigments with 80% acetone at 4 °C for 48 h under dark condition. Then, contents of chlorophylls (Chl) and carotenoids (Caro) were measured using a BIOMATE 35 UV-Visible Spectrophotometer (Thermo Scientific) at 470 nm, 646 nm and 663 nm, and were calculated according to the method of Lichtenthaler and Wellburn (1983). The pigment data of each sample were measured for three independent experiment repeats.

### Measurement of Net Photosynthetic Rate

Net photosynthetic rate (Pn) was measured under 400 ppm of CO_2_ concentration and sunny weather condition, during 09:30–10:30 a.m. when the solar radiation was approximately 1200 μmol m^− 2^ s^− 1^. During grain-filling stage, the flag leaves of wild type and *ys53* mutant was selected to measure the photosynthetic capability with a portable photosynthetic apparatus (Li-6400, Li-COR Inc., USA) (Sun et al. 2012).

### Map-Based Cloning and Marker Development

Eight hundred fifty-two recessive plants which showed yellow-leaf phenotype were selected from the (*ys53*/Minghui 63) F_2_ segregating population. The whole genome DNA of *ys53*, Minghui 63 and these recessive plants were separately extracted for the linkage mapping analysis based on simple sequence repeat (SSR) markers. The SSR markers used were filtered from the Gramene database (http://archive.gramene.org/markers/microsat/) of the SSR linkage map constructed by McCouch et al. (2002). For fine mapping, the insertion/deletion (InDel) markers were selected, which were based on the flanking sequence that contains natural difference of 25–75 bp of gap between *japonica* rice cv Nipponbare and *indica* rice cv 9311 in the multiple sequence alignment, and designed by Primer 5.0. All BLAST searches of the markers were performed by using National Center for Biotechnology Information database (http://www.ncbi.nlm.nih.gov/BLAST/).

### Transmission Electron Microscopy Analysis

The fully expanding leaves were harvested from the *ys53* mutant and its wild type Nipponbare at the three-leaf stage. The selected leaf tissues were fixed by soaking in a phosphate buffer containing 3% glutaraldehyde and then fixed again with 1% osmium dioxide. Next, the sample was gradually dehydrated, embedded, sectioned, and stained. Finally, submicroscopic structure of the sample was observed by transmission electron microscopy (H-600IV, Hitachi) (Wang et al. 2010).

### Sequence Analysis

The full-length cDNA and amino acid sequences of OsGSAM and its homologues were acquired from GeneBank (http://www.ncbi.nlm.nih.gov). The chloroplast signal peptide was predicted according to http://www.cbs.dtu.dk/services/TargetP (Emanuelsson et al. 2007). Multiple sequence alignment was performed using DNAMAN version 6.0 (Lynnon Biosoft). The phylogenetic tree was constructed using the program MEGA 7.0 (Mega Limited) and the maximum likelihood algorithm.

### Determination of ALA and GSA Contents and ALA-Synthesis Ability

In order to measure total contents of ALA and GSA under natural condition, the fresh leaves of rice were collected from normal growing plants at seedling stage in the paddy field on sunny afternoons, and immediately stored in liquid nitrogen environment. The detection of ALA and GSA contents was performed according to the protocol of Mauzerall and Granick (1955) with slight optimization. First, the leaf tissues were homogenated adequately in 5 ml of 4% trichloroacetic acid (TCA, PH 1.0). After centrifugation for 5 min at 8000 r/min and 4 °C, 1 mL of the extraction solution was taken into a new tube, and 500 μL 1 M NaAc and 50 μL acetylacetone were added into this tube. Then, the mixed reaction solution in the tube was cooled to room temperature after 10 min in a boiling water bath and centrifuged at 8000 r/min for 5 min. Subsequently, 1.0 ml of the supernatant was taken in another new tube, and an equal volume of Ehrlich’s reagent was added into this tube for reaction under dark condition for 10 min. Finally, the absorbance of the sample was measured at 553 nm, and the ALA and GSA concentration can be obtained according to the standard curve.

For the determination of ALA-synthesising activity, the four steps, including acquisition of yellow seedlings, accumulation, extraction and determination of ALA, were successively carried out. First, the acquisition of yellow seedlings and accumulation of ALA were performed according to the method described previously (Beale 1970; Aarti et al. 2007) with a slight modification. Seeds of *ys53* and its wild type were cultivated for 1 week in the dark, and 0.4 g young leaf tissues were harvested and immersed into 100 ml of 20 mM LA (levulinic acid) solution under 7 h of light (100 μmol m^− 2^ s^− 1^) for ALA accumulation. Then, the leaf tissues were homogenated adequately in 5 ml of 50 mM phosphate buffer (pH 6.8). Subsequently, extraction and determination of ALA are performed using the above-mentioned method.

### Complementation Analysis

For complementation of *ys53*, the full-length cDNA sequence (1437 bp) of *OsGSAM* (*LOC_Os08g41990*) was amplified from the wild-type Nipponbare using a pair of primers 5′-GAGTCTAGAATGGCCGGAGCAGCAGCC-3′ and 5′-TCGCTGCAGCTATATCCGGCGAAGAAC-3′, which have a *Xba*I site at 5′-end and a *Pst*I site at 3′-end of this gene respectively. After digested with enzymes *Xba*I and *Pst*I, the PCR product was ligated into the binary vector pCAMBIA2300. The constructed pCAMBIA2300-*OsGSAM* vector, which contained the target *OsGSAM* gene driven by the *Actin 1* promoter, was transferred into the *ys53* mutant by *Agrobacterium tumefaciens*-mediated transformation. Transgenic plants were detected by a pair of primers 5′-CTTATGGTGGGGCTCAAG-3′ and 5′-GCGATCATAGGCGTCTCG-3′ which located on the *OsGSAM* gene and the pCAMBIA2300 vector respectively (Additional file 1: Supplementary Table S5).

### Subcellular Localization

The full-length cDNA fragment of *OsGSAM* was amplified from the wild type and inserted into pCAMBIA2300-35S-eGFP vector. The PCR primers were 5′-GAGGGATCCATGGCCGGAGCAGCAGCC-3′ and 5′-TCGTCTAGACTCTATCCGGCGAAGAAC-3′, which contained a *Bam*HI site at the 5′-end and a *Xba*I site at the 3′-end of the cDNA fragment respectively (Additional file 1: Supplementary Table S5). The constructed fusion vector pCAMBIA2300-35S-*OsGSAM*-eGFP and the empty vector pCAMBIA2300-35S-eGFP (negative control) were transformed into the rice protoplasts respectively, following the method as previously described (Zhang et al. 2011). Finally, the GFP fluorescence in the transformed protoplasts was examined under a laser-scanning confocal microscopy (Nikon A1).

### qRT–PCR Analysis

The roots, stems, leaf sheaths, leaf blades and young panicles of rice in different stages were harvested from the naturally growing plants of *ys53* and its wild type in the field at early morning, and the total RNA was extracted with RNA extraction kit (Vazyme). The first strand of cDNA is reversed-transcribed from total RNA (2 μg) using a reverse transcription kit (Vazyme) according to the manufacturer’s instructions. The primer pair qGSAM-1F (5′-TGGACGTAAGGACATCAT-3′) and qGSAM-1R (5′-GTCCAAGTAATCGTAGGT-3′) were used to quantify the *OsGSAM* expression levels of the various samples to describe the expression pattern of the gene. To measure the relative expression of the genes associated with tetrapyrrole biosynthesis and photosynthesis, 22 pairs of primers were developed. Real-time quantitative PCR (qRT-PCR) was performed in a total volume of 10 μL containing 0.2 μM of each primer pairs and 1 × SYBR green PCR master mix (Vazyme) by using the CFX96 real-time PCR system (Bio-Rad). All reaction conditions were as follows: 95 °C for 5 min, then 40 cycles of 95 °C for 10 s and 58 °C for 30 s. For each experimental group, qRT-PCR was operated with three technical replicates for each of three biological replicates. The 2^-△△CT^ method was applied to calculate the quantitative expression of each genes relative to the internal control. The *Actin 1* gene was used for normalization as an internal control. All qRT-PCR primers were listed in Additional file 1: Supplementary Table S4.

### Yeast Two Hybrid Assay

The Matchmaker GAL4 two-hybrid system 3 of Clontech (Clontech, USA) was used. The full-length cDNA and a series of truncated cDNA sequences of *OsGSAM* gene were amplified and inserted into pGADT7 and pGBKT7 vectors respectively, and the *Bam*HI site in the vectors was chosen as the insertion site for all sequences. The self-activation was tested by the bait construct fused with BD. The combination containing the self-activating fusion vector was supplemented with 30 mM 3-AT (3-amino-1,2,4-triazole) in its corresponding medium. The above recombinant vectors were combined according to experimental needs, and then co-transformed into the yeast strain Y2HGold cells. Each transformant was spotted on non-selective (−Leu/−Trp) plates for 3 d at 28 °C, and tested for the protein interaction by observing their growth situation on selective (−Ade/−His/−Leu/−Trp) plates. The saturated culture solution of each transformant was diluted to concentrations of 1, 0.1 and 0.01, and dripped onto its corresponding plates. Interaction between pGADT7-T and pGBKT7-Lam was used as a negative control, and interaction between pGADT7-T and pGBKT7–53 was used as a positive control. The pGBKT7 vector fused with full-length cDNA sequence was combined with empty pGADAD vecter as internal control. Primer sets used for generating constructs were listed in Additional file 1: Supplementary Table S6.

## Supplementary Information

**Additional file 1:** **Table S1.** Segregation of F_2_ population from the cross between *ys53* and normal green leaf variety. **Table S2.** Putative genes within the 102.5-kb region. **Table S3.** The sequenced genes and corresponding PCR amplification primers in the fine mapping region. **Table S4.** Primers used in qRT-PCR. **Table S5.** Primers used in vector construction for complementation and subcellular localization. **Table S6.** Primers used in OsGSAM truncation experiment.

**Additional file 2:** **Fig. S1.** Internode length of *ys53* and its wild type. **Fig. S2.** Temperature treatment of the *ys53* mutant and its wild type. **Fig. S3.** Sequence alignment of OsGSAM and its homologues. **Fig. S4.** Phylogenetic analysis of OsGSAM and its homologs. **Fig. S5.** Tetrapyrrole biosynthetic pathway in higher plants.

## Data Availability

All data generated or analyzed during this study are included in this article (and its supplementary information files).

## Abbreviations

ALA: 5-aminolevulinic acid
GSA: Glutamate 1-semialdehyde
GSAM: Glutamate 1-semialdehyde 2,1-aminomutase
Chl: Chlorophyll
GFP: Green fluorescent protein
Y2H: Yeast two hybrid
