# Supplementary material for: A Single Nucleotide Substitution of GSAM Gene Causes Massive Accumulation of Glutamate 1-Semialdehyde and Yellow Leaf Phenotype in Rice
Source: Rice (N Y). 2021 Jun 5;14:50. doi: 10.1186/s12284-021-00492-x (PMC8179877; doi:10.1186/s12284-021-00492-x)
Supplement: Supplementary file 1 — Additional file 1: Table S1. Segregation of F2 population from the cross between ys53 and normal green leaf variety. Table S2. Putative genes within the 102.5-kb region. Table S3. The sequenced genes and corresponding PCR amplification primers in the fine mapping region. Table S4. Primers used in qRT-PCR. Table S5. Primers used in vector construction for complementation and subcellular localization. Table S6. Primers used in OsGSAM truncation experiment. [file 12284_2021_492_MOESM1_ESM.doc]

**Supplementary Table S1** Segregation of F_2_ populations from the crosses between *ys53* and normal green leaf variety.

| Combination | No. of total plants | No. of green leaf plants | No. of yellow  leaf plants | Expected ratio | χ^2^ | χ^2^_0.05_ |
| --- | --- | --- | --- | --- | --- | --- |
| *ys53* /Minghui 63 | 3440 | 2588 | 852 | 3:1 | 0.09 | 3.84 |

**Supplementary Table S2** Putative genes within the 102.5-kb region.

| Number | MSU Locus ID | Putative functions |
| --- | --- | --- |
| ORF1 | *LOC_Os08g41940* | Osspl16-SBP-box gene family member, expressed |
| ORF2 | *LOC_Os08g41950* | Osmads7-MADS-box family gene with mikcc type-box, expressed |
| ORF3 | *LOC_Os08g41960* | Osmads37-MADS-box family gene with MIKC* type-box, expressed |
| ORF4 | *LOC_Os08g41980* | Expressed protein |
| ORF5 | *LOC_Os08g41990* | Aminotransferase, putative, expressed |
| ORF6 | *LOC_Os08g42000* | Nuclear transport factor, putative, expressed |
| ORF7 | *LOC_Os08g42010* | Nodulin, putative, expressed |
| ORF8 | *LOC_Os08g42020* | Zinc ion binding protein, putative, expressed |
| ORF9 | *LOC_Os08g42030* | Peroxidase precursor, putative, expressed |
| ORF10 | *LOC_Os08g42040* | LTPL80-Protease inhibitor/seed storage/LTP family protein precursor, expressed |
| ORF11 | *LOC_Os08g42050* | Emp24/gp25l/p24 family protein, putative, expressed |
| ORF12 | *LOC_Os08g42060* | Expressed protein |
| ORF13 | *LOC_Os08g42080* | ACR5, putative, expressed |
| ORF14 | *LOC_Os08g42090* | Retrotransposon protein, putative, Ty1-copia subclass, expressed |

**Supplementary Table S3** The sequenced genes and corresponding PCR amplification primers in the fine mapping region.

| ID | Primers Name | Forward primer (5′-3′) | Reverse primer (5′-3′) |
| --- | --- | --- | --- |
| *LOC_Os08g41990* | 1F/1R  2F/2R  3F/3R | GACCGGTGGACCCATGCG  CTCGTATGTGGGATGTGG  AGTCCGAGGAGATCTTCA | GTACCATGGTTTGGATTC  CATGGATGCTACTAACTC  GCTCACATTGTCCAACTC |
| *LOC_Os08g42000*  *LOC_Os08g42010*  *LOC_Os08g42020*  *LOC_Os08g42030*  *LOC_Os08g42040*  *LOC_Os08g42050*  *LOC_Os08g42060*  *LOC_Os08g42080* | 1F/1R  2F/2R  1F/1R  2F/2R  3F/3R  4F/4R  1F/1R  2F/2R  1F/1R  2F/2R  1F/1R  2F/2R  1F/1R  2F/2R  1F/1R  1F/1R  2F/2R | CTGTCCAATGGGTTAGCG  GCTCTCGTTAATCTAATG  CGGCTCGTGCTGGATTAC  CAGTATCTGTGAAGGATAG  GATGCTCTAGTCGAGTGC  CTACAGTAGCTGTTTACTG  GTATTCGTGACTCGGTTG  GCTCCTGAACGCACCCGTG  TCACCTCTCACACCACTGCT  TGTTACTAGTTCAGCGAGTG  ATCGCCACCGTCGTTCAC  CCTACTCTTGGAACGAGG  CTTATGGTTGATGTTTGCGC  ACATCGCTTCTGCTTCAC  CTCACTCTCCAAACGTGC  CAGCAAGTAGTACTATACAC  CCAGATGATGCACGACGACC | CCACTGACATGCCAACAAC  CAAATCCAGAAGGTGCATAG  CCCATATCCCATACACGG  CTGTTGAGAGATGAGATG  CATCAGATGGATCACAAAG  CTTCACCCTTCTGCTCTC  CATCATCAACGGCTGAGC  CTTCACCAAAGTGGTACTC  CTCTACGTGCATTCACGCGC  GTATTACACTCTTCAGTCTG  GAAGAGGAGTTATAGAGC  GTACAACATCAGAACGAATC  GAGATGGCAGTAACTTATAG  GTCTATGTGACAGCAACC  GAGAGTGGCTATCTCACTC  GTGCACACGGTGTCGAACAG  CATGGATCAACGAACACGAC |

**Supplementary Table S4** Primers used in qRT-PCR.

| Marker | Forward primer (5′-3′) | Reverse primer (5′-3′) | Reference |
| --- | --- | --- | --- |
| *HEMA*  *GSAM*  *HEMB*  *CHLD*  *CHLH*  *CHLI*  *CHLM*  *CHL27* | CGCTATTTCTGATGCTATGGGT  TGGACGTAAGGACATCAT  ACCTGGATTGCCATACTT  CAAGGGTCGCCCAAGGTAAA  CCAATCCGTAACCCGAAGGT  TTCGACAGGGATCCAAAGGC  GGCTTCATCTCCACGCAGTT  CCGCAGGCTCAAGAAAAC | TCTTGGGTGATGATTGTTTGG  GTCCAAGTAATCGTAGGT  TTCGTCGATCATGTTCAGTG  CTTCAGGTCCGAGAGTGCAG  CAATAATTTTGGCGCTCTTCAA  ACAGCACCAAGGTTACTCCG  GACGAATCGAAGACGCACAA  ATCAGACAGCCCCTTGTTCA | Su et al., 2012  Li et al., 2019  Inagaki et al., 2015  Li et al., 2019  Li et al., 2019  Inagaki et al., 2015 |
| *DVR* | CCATTGCCAGTTTCTTGGTG | AATTGAATGGCTAATGGCGT | Inagaki et al., 2015 |
| *PORA*  *YGL1* | TGTACTGGAGCTGGAACAACAA  GGCACTGCTAGGACTCAC | GAGCACAGCAAAATCCTAGACG  CCCAAGACGAAGAACGGT | Su et al., 2012  Wu et al. 2007 |
| *CAO1* | GATCCATACCCGATCGACAT | CGAGAGACATCCGGTAGAGC | Su et al., 2012 |
| *FC1* | GGTCAACAGGGTGTAAAGAG | GTGCATCCAAGAGCTGGAAC | Inagaki et al., 2015 |
| *FC2* | CTTGCCTTATGTTGGTGCTA | AGCCCCACTCCCATACAGTC | Inagaki et al., 2015 |
| *rbcS* | CAGCAATGGCGGCAGGAT | AGGGCACCCACTTGGAACG | Li et al., 2015 |
| *rbcL* | CTTGGCAGCATTCCGAGTAA | ACAACGGGCTCGATGTGATA | Su et al., 2012 |
| *psaA* | GGAGGTGGCGAGTTAGTA | GATTTGCTTTATCGGGTAT | Li et al., 2015 |
| *psbA* | TATGGGTCGTGAGTGGGA | TTATGCTCTGCCTGGAAT | Li et al., 2015 |
| *psaN* | CTGAACGACAAGAAGAGG | CACCA TTTCCAGAAGACG | Huang et al., 2017 |
| *psbP* | TCAACACTCAGCAAGCAG | ATCCTGGTATGGAGGAAC | Huang et al., 2017 |
| *CAB1R* | AGA TGGGTTTAGTGCGACGAG | TTTGGGA TCGAGGGAGTATTT | Su et al., 2012 |
| *CAB2R* | TGTTCTCCA TGTTCGGCTTCT | GCCCAGGCGTTGTTGTTGA | Su et al., 2012;  Ma et al., 2017 |
| *Actin 1* | TGTATGCCAGTGGTCGTACCA | CCAGCAAGGTCGAGACGAA | Li et al., 2015 |

Inagaki N, Kinoshita K, Kagawa T, Tanaka A, Ueno O, Shimada H and Takano M (2015) Phytochrome B mediates the regulation of chlorophyll biosynthesis through transcriptional regulation of *ChlH* and *GUN4* in rice seedlings. PLoS ONE 10:e0135408

Huang R, Wang Y, Wang PR, Li CM, Xiao FL, Chen NG, Li N, Li CX, Sun CH, Li LH, Chen RJ, Xu ZJ, Zhu JQ and Deng XJ (2018) A single nucleotide mutation of *IspF* gene involved in the MEP pathway for isoprenoid biosynthesis causes yellow-green leaf phenotype in rice. Plant Mol Biol 96:5–16

Li CM, Hu Y, Huang R, Ma XZ, Wang Y, Liao TT, Zhong P, Xiao FL, Sun CH, Xu ZJ, Deng XJ and Wang PR (2015) Mutation of *FdC2* gene encoding a ferredoxin-like protein with C-terminal extension causes yellow-green leaf phenotype in rice. Plant Sci 238:127–134

Li CM, Liu X, Pan JH, Guo J, Wang Q, Chen CP, Li N, Zhang K, Yang B, Sun CH, Deng XJ and Wang PR (2019) A *lil3 chlp* double mutant with exclusive accumulation of geranylgeranyl chlorophyll displays a lethal phenotype in rice. BMC Plant Biol 19:456–470

Ma XZ, Sun XQ, Li CM, Huang R, Sun CH, Wang Y, Xiao FL, Wang Q, Chen PR, Ma FR, Zhang K, Wang PR and Deng XJ (2017) Map-based cloning and characterization of the novel yellow-green leaf gene *ys83* in rice (*Oryza sativa*). Plant Physiol Biochem 111:1–9

Su N, Hu ML, Wu DX, Wu FQ, Fei GL, Lan Y, Chen XL, Shu XL, Zhang X, Guo XP, Cheng ZJ, Lei CL, Qi CK, Jiang L, Wang HY and Wan JM (2012) Disruption of a rice pentatricopeptide repeat protein causes a seedling-specific albino phenotype and its utilization to enhance seed purity in hybrid rice production. Plant Physiol 159:227–238

Wu ZM, Zhang X, He B, Diao LP, Sheng SL, Wang JL, Guo XP, Su N, Wang LF, Jiang L, Wang CM, Zhai HQ and Wan JM (2007) A chlorophyll-deficient rice mutant with impaired chlorophyllide esterification in chlorophyll biosynthesis. Plant physiol 145:29–40

**Supplementary Table S5** Primers used in vector construction for complementation and subcellular localization.

| Primer name | Primer pairs (5′-3′) | Restriction enzyme |
| --- | --- | --- |
| GSAM-CM-1F | GAGTCTAGAATGGCCGGAGCAGCAGCC | *Xba*I |
| GSAM-CM-1R  GSAM-SL-1F  GSAM-SL-1R | TCGCTGCAGCTATATCCGGCGAAGAAC  GAGGGATCCATGGCCGGAGCAGCAGCC TCGTCTAGACTCTATCCGGCGAAGAAC | *Pst*I  *Bam*HI  *Xba*I |

**Supplementary Table S6** Primers used in OsGSAM truncation experiment.

| Fragment  name | Primer name | Primer pairs (5′-3′) |
| --- | --- | --- |
| **FL** | GSAM^1-478^-AD-F | GTGGGCATCGATACGGGATCCATATGGCCGGAGCAGCAGCC |
| **FL** | GSAM^1-478^-AD-R | CAGCTCGAGCTCGATGGATCCCTATATCCGGCGAAGAAC |
| **FL** | GSAM^1-478^-BD-F | AGGCCGAATTCCCGGGGATCCGTATGGCCGGAGCAGCAGCC |
| **FL** | GSAM^1-478^-BD-R | CCGCTGCAGGTCGACGGATCCCTATATCCGGCGAAGAAC |
| **R1** | GSAM^1-240^-AD-F | GTGGGCATCGATACGGGATCCATATGGCCGGAGCAGCAGCC |
| **R1** | GSAM^1-240^-AD-R | CAGCTCGAGCTCGATGGATCCTGCCTCGACATCATTGTA |
| **R1** | GSAM^1-240^-BD-F | AGGCCGAATTCCCGGGGATCCGTATGGCCGGAGCAGCAGCC |
| **R1** | GSAM^1-240^-BD-R | CCGCTGCAGGTCGACGGATCCTGCCTCGACATCATTGTA |
| **R2** | GSAM^241-478^-AD-F | GTGGGCATCGATACGGGATCCATGTGAAAAAACTGTTTGAG |
| **R2** | GSAM^241-478^-AD-R | CAGCTCGAGCTCGATGGATCCCTATATCCGGCGAAGAAC |
| **R2** | GSAM^241-478^-BD-F | AGGCCGAATTCCCGGGGATCCGTGTGAAAAAACTGTTTGAG |
| **R2** | GSAM^241-478^-BD-R | CCGCTGCAGGTCGACGGATCCCTATATCCGGCGAAGAAC |
| **R3** | GSAM^80-400^-AD-F | GTGGGCATCGATACGGGATCCATTCAGTTGGTGGGCAGCCC |
| **R3** | GSAM^80-400^-AD-R | CAGCTCGAGCTCGATGGATCCACACATCTCATGTCCAGT |
| **R3** | GSAM^80-400^-BD-F | AGGCCGAATTCCCGGGGATCCGTTCAGTTGGTGGGCAGCCC |
| **R3** | GSAM^80-400^-BD-R | CCGCTGCAGGTCGACGGATCCACACATCTCATGTCCAGT |
| **R4** | GSAM^161-320^-AD-F | GTGGGCATCGATACGGGATCCATATGGTCCGTTTTGTCAAT |
| **R4** | GSAM^161-320^-AD-R | CAGCTCGAGCTCGATGGATCCGATGATTTTCCCCAATGT |
| **R4** | GSAM^161-320^-BD-F | AGGCCGAATTCCCGGGGATCCGTATGGTCCGTTTTGTCAAT |
| **R4** | GSAM^161-320^-BD-R | CCGCTGCAGGTCGACGGATCCGATGATTTTCCCCAATGT |
| **R5** | GSAM^1-160^-AD-F | GTGGGCATCGATACGGGATCCATATGGCCGGAGCAGCAGCC |
| **R5** | GSAM^1-160^-AD-R | CAGCTCGAGCTCGATGGATCCTTCGATACTTGGTACAGC |
| **R5** | GSAM^1-160^-BD-F | AGGCCGAATTCCCGGGGATCCGTATGGCCGGAGCAGCAGCC |
| **R5** | GSAM^1-160^-BD-R | CCGCTGCAGGTCGACGGATCCTTCGATACTTGGTACAGC |
| **R6** | GSAM^321-478^-AD-F | GTGGGCATCGATACGGGATCCATGGTGGCGGTCTTCCAGTT |
| **R6** | GSAM^321-478^-AD-R | CAGCTCGAGCTCGATGGATCCCTATATCCGGCGAAGAAC |
| **R6** | GSAM^321-478^-BD-F | AGGCCGAATTCCCGGGGATCCGTGGTGGCGGTCTTCCAGTT |
| **R6** | GSAM^321-478^-BD-R | CCGCTGCAGGTCGACGGATCCCTATATCCGGCGAAGAAC |
| **R7** | GSAM^1-80^-AD-F | GTGGGCATCGATACGGGATCCATATGGCCGGAGCAGCAGCC |
| **R7** | GSAM^1-80^-AD-R | CAGCTCGAGCTCGATGGATCCTTTGAAGGCACGAACTGG |
| **R7** | GSAM^1-80^-BD-F | AGGCCGAATTCCCGGGGATCCGTATGGCCGGAGCAGCAGCC |
| **R7** | GSAM^1-80^-BD-R | CCGCTGCAGGTCGACGGATCCTTTGAAGGCACGAACTGG |
| **R8** | GSAM^81-160^-AD-F | GTGGGCATCGATACGGGATCCATTCAGTTGGTGGGCAGCCC |
| **R8** | GSAM^81-160^-AD-R | CAGCTCGAGCTCGATGGATCCTTCGATACTTGGTACAGC |
| **R8** | GSAM^81-160^-BD-F | AGGCCGAATTCCCGGGGATCCGTTCAGTTGGTGGGCAGCCC |
| **R8** | GSAM^81-160^-BD-R | CCGCTGCAGGTCGACGGATCCTTCGATACTTGGTACAGC |
| **R9** | GSAM^321-400^-AD-F | GTGGGCATCGATACGGGATCCATGGTGGCGGTCTTCCAGTT |
| **R9** | GSAM^321-400^-AD-R | CAGCTCGAGCTCGATGGATCCACACATCTCATGTCCAGT |
| **R9** | GSAM^321-400^-BD-F | AGGCCGAATTCCCGGGGATCCGTGGTGGCGGTCTTCCAGTT |
| **R9** | GSAM^321-400^-BD-R | CCGCTGCAGGTCGACGGATCCACACATCTCATGTCCAGT |
| **R10** | GSAM^400-478^-AD-F | GTGGGCATCGATACGGGATCCATGGAGGACACATCAGGGGG |
| **R10** | GSAM^400-478^-AD-R | CAGCTCGAGCTCGATGGATCCCTATATCCGGCGAAGAAC |
| **R10** | GSAM^400-478^-BD-F | AGGCCGAATTCCCGGGGATCCGTGGAGGACACATCAGGGGG |
| **R10** | GSAM^400-478^-BD-R | CCGCTGCAGGTCGACGGATCCCTATATCCGGCGAAGAAC |
