# Supplementary material for: A Single Nucleotide Substitution of GSAM Gene Causes Massive Accumulation of Glutamate 1-Semialdehyde and Yellow Leaf Phenotype in Rice
Source: Rice (N Y). 2021 Jun 5;14:50. doi: 10.1186/s12284-021-00492-x (PMC8179877; doi:10.1186/s12284-021-00492-x)
Supplement: Supplementary file 2 — Additional file 2: Fig. S1. Internode length of ys53 and its wild type. Fig. S2. Temperature treatment of the ys53 mutant and its wild type. Fig. S3. Sequence alignment of OsGSAM and its homologues. Fig. S4. Phylogenetic analysis of OsGSAM and its homologs. Fig. S5. Tetrapyrrole biosynthetic pathway in higher plants. [file 12284_2021_492_MOESM2_ESM.doc]

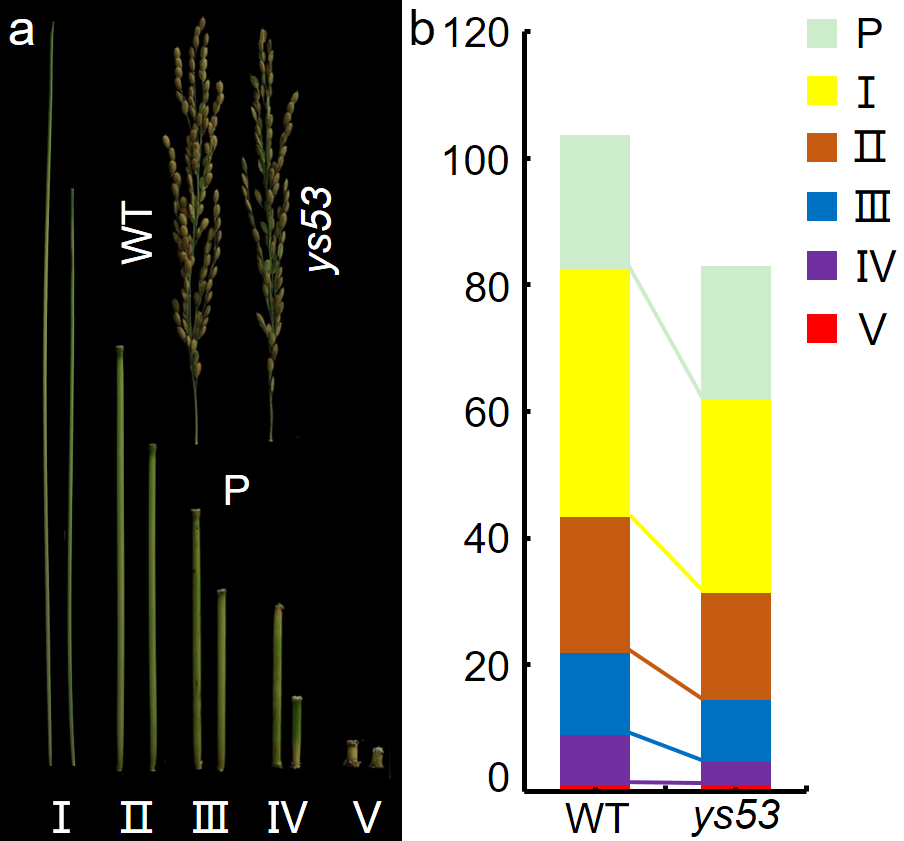


**Supplementary Figure S1** Internode length of *ys53* and its wild type. **a** The panicle and internode phenotypes of *ys53* and its wild type. **b** The panicle and internode lengths of *ys53* and its wild type.


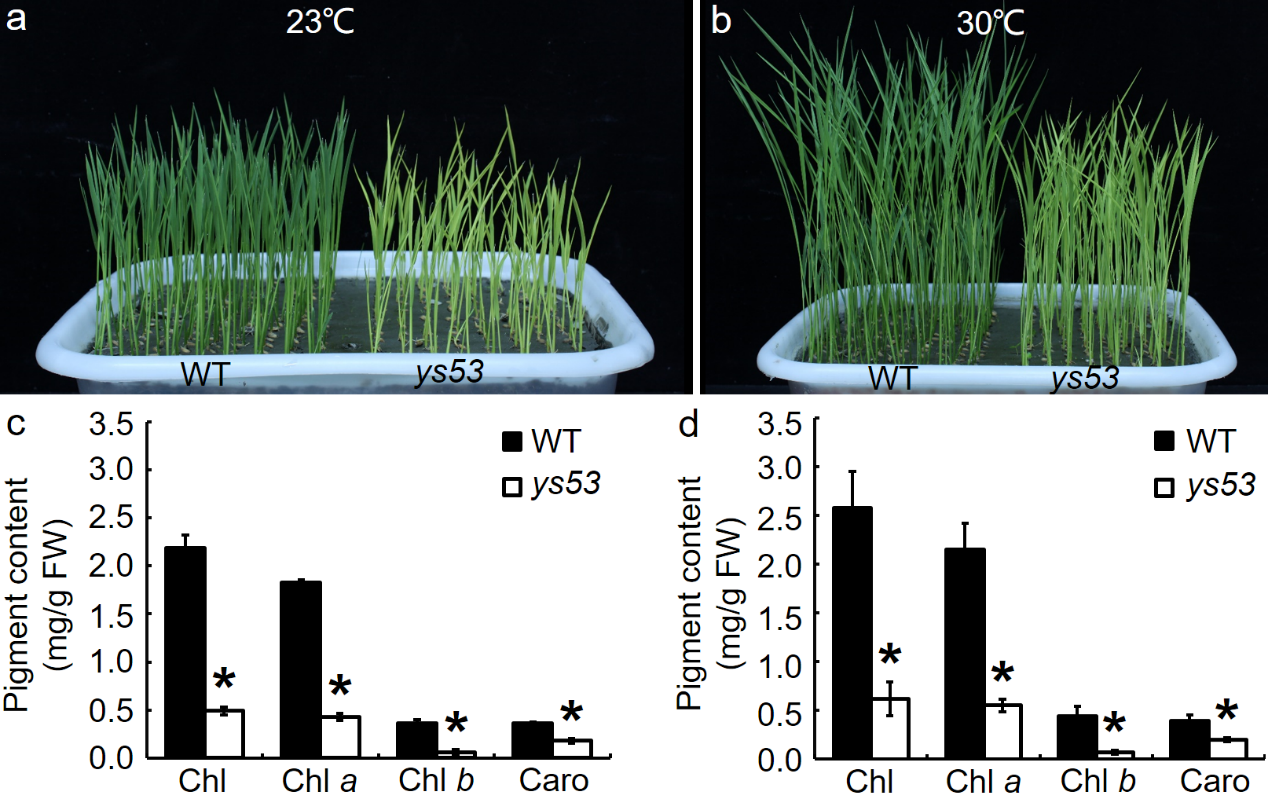


**Supplementary Figure S2** Temperature treatment of the *ys53* mutant and its wild type. **a** and **b** Seedlings at three-leaf stage in 23℃ and 30℃, respectively. **c** and **d** Pigment contents at three-leaf stage in 23℃ and 30℃, respectively. Both the mutant and its wild type were grown in the growth chamber under 12 h of light (80-100 µmol m^-2^ s^-1^)/12 h of dark at constant 23°C (low temperature) or 30°C (high temperature). Error bars represent the SDs of three independent experiments. * indicate statistically significant differences between *ys53* and its wild type at *P* < 0.05.


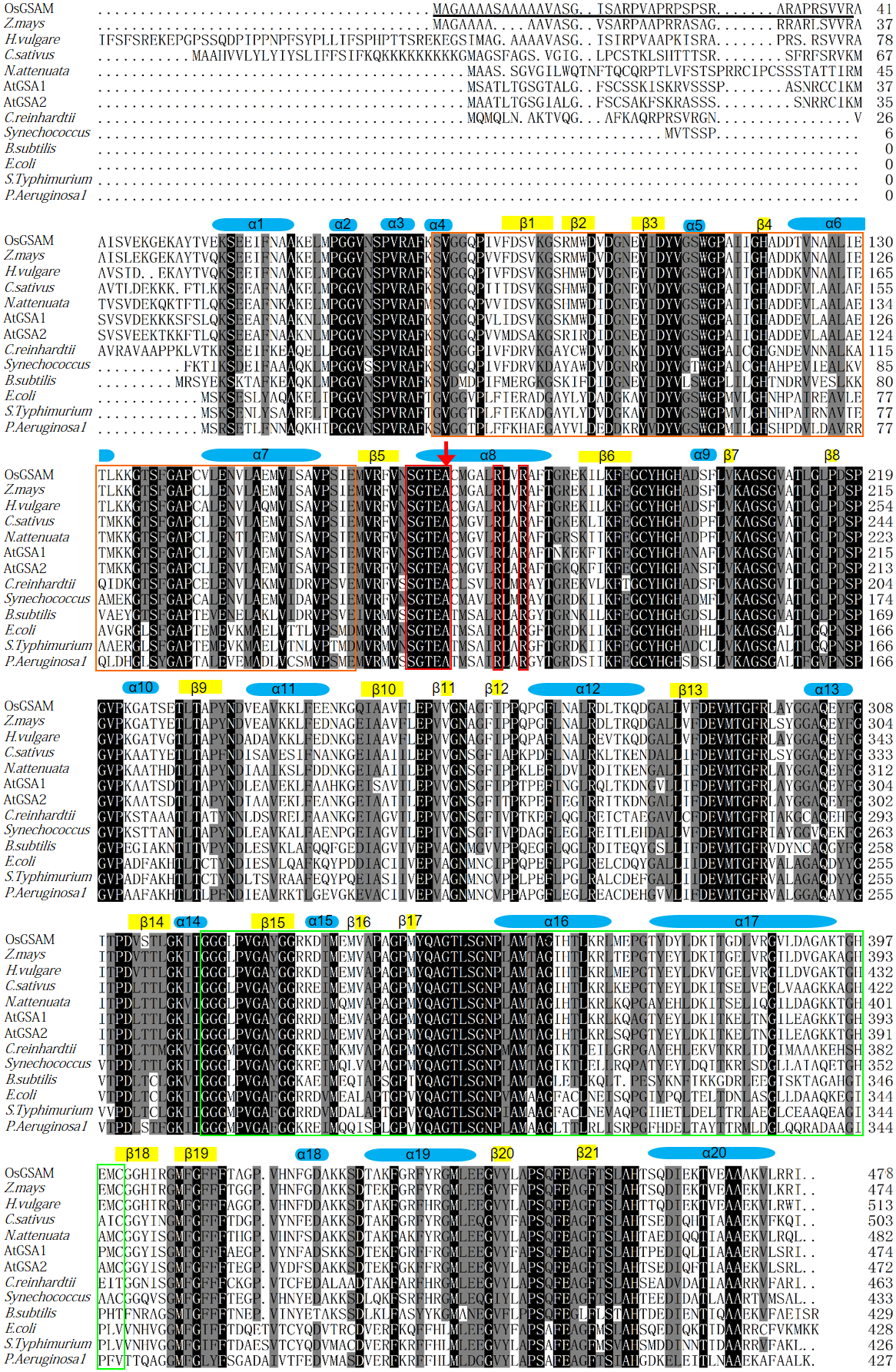


**Supplementary Figure S3** Sequence alignment of OsGSAM and its homologues. Identical residues are shown on a black background, and similar residues (≥75% identical) are shown on a gray background. The putative chloroplast signal peptide is indicated by a black underline (1^th^-40^th^ amino acid residues). The light blue block and the yellow square indicate α helix and β sheet of OsGSAM, respectively. The orange frame and the green frame is the protein interaction regions R8 (N-ends) and R9 (C-ends) identified in this study, respectively. The red arrow indicates the amino acid substitution from Ala-171 to Thr in the *ys53* mutant. The red frame indicates the interface helix residues in *Synechococcus*. GenBank accession numbers for the respective protein sequences are as follows: *Oryza Sativa* (OsGSAM, LOC_Os08g41990); *Zea mays* (NP_001345686.1); *Hordeum vulgare* (KAE8796537.1); *Arabidopsis thaliana* AtGSA2 (AT3G48730, NP_190442.1) and AtGSA1 (AT5G63570, NP_201162.1); *Nicotiana attenuata* (XP_019224273.1); *Cucumis sativus* (XP_004136907.3); *Synechococcus* (ABL10417.1); *Chlamydomonas reinhardtii* (XP_001697519.1); *Bacillus subtilis* (WP_004398699.1); *Escherichia coli* (WP_139963501.1); *Salmonelta typhimurium* (WP_000045268.1); *Pseudomonas aeruginosa* (WP_003093150.1).


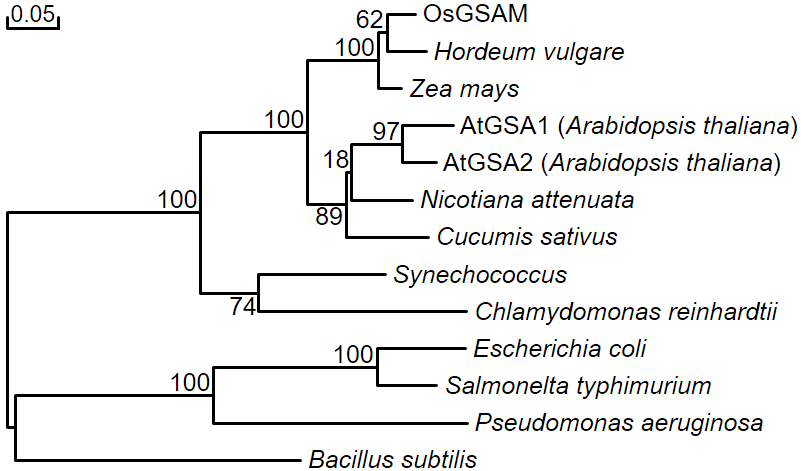


**Supplementary Figure S4** Phylogenetic analysis of OsGSAM and its homologs. The phylogenetic tree was constructed by the program MEGA 7, and calculated by the maximum likelihood algorithm. Bootstrap support based on 1000 replicates was shown at each node. Scale bar represents the average number of substitutions per residues. GenBank accession numbers for the respective protein sequences are as Supplemental Figure S3.


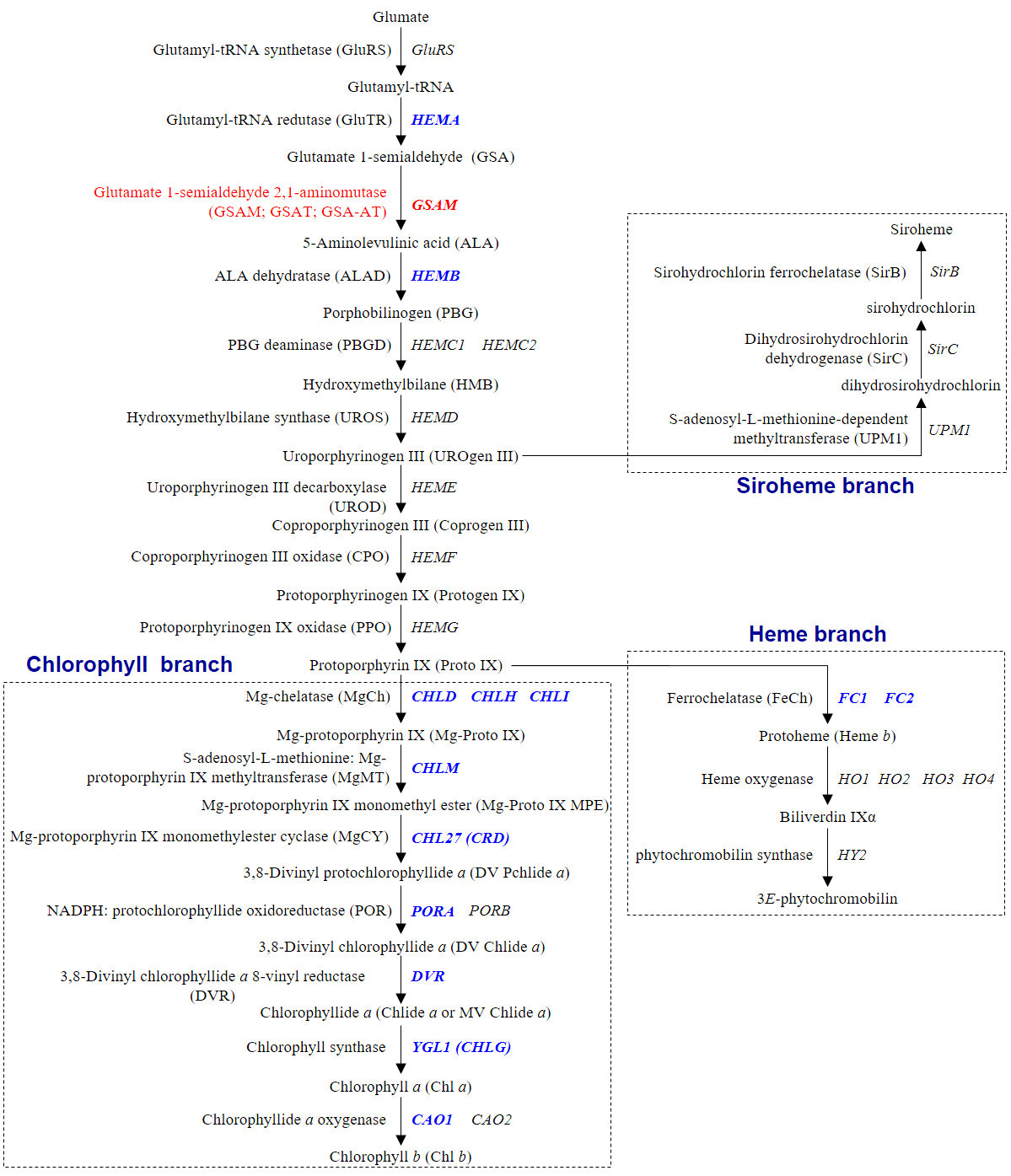


**Supplementary Figure S5** Tetrapyrrole biosynthetic pathway in higher plants. The italicized texts indicate genes encoding the corresponding enzyme proteins on the left, and the blue texts indicate the genes analyzed using the qRT-PCR in the present study. The red texts indicate the mutant gene *GSAM* and its encoded enzyme in this study.
